# Medical diagnosis as a linguistic game

**DOI:** 10.1186/s12911-017-0488-3

**Published:** 2017-07-10

**Authors:** Peter Fritz, Andreas Kleinhans, Florian Kuisle, Patricius Albu, Christine Fritz-Kuisle, Mark Dominik Alscher

**Affiliations:** 1 Department of Clinical Pathology, Robert-Bosch-Hospital, Stuttgart, Germany; 2 Department of General Internal Medicine and Nephrology, Robert-Bosch-Hospital, Auerbachstrasse 110, D-70376 Stuttgart, Germany; 3 Klinikum Günzburg, Abteilung für Anästhesie, Günzburg, Germany

**Keywords:** Formalized medical knowledge, Medical data base, Linguistic game, Disease entity

## Abstract

**Background:**

We present a formalized medical knowledge system using a linguistic approach combined with a semantic net.

**Method:**

Diseases are defined and coded by natural linguistic terms and linked via a complex network of attributes, categories, classes, lists and other semantic conditions.

**Results:**

We have isolated more than 4600 disease entities (termed pathosoms using a made-up word) with more than 100.000 attributes sets (termed pathophemes using a made-up word) and a semantic net with more than 140.000 links. All major-medical thesauri like ICD, ICD-O and OPS are included.

**Conclusions:**

Memem7 is a linguistic approach to medical knowledge approach. With the system, we performed a proof of concept and we conclude from our data that our or similar approaches provides reliable and feasible tools for physicians given a formalized history taking is available. Our approach can be considered as both a linguistic game and a third opinion to a set of patient’s data.

**Electronic supplementary material:**

The online version of this article (doi:10.1186/s12911-017-0488-3) contains supplementary material, which is available to authorized users.

## Background

Medicine involves doctors and patients discussing risk and reaching complex decisions together about treatment options, amongst other things. Yet it is known that diagnostic errors occur, with a frequency (depending on how terms are defined) ranging between 2.5% and 20% [1, 2]. Most errors have only moderate negative consequences, but some do not. Modern computing technology is claimed to decrease the number of erroneous diagnosis [3–5]. Nowadays, numerous systems of collecting patient data (electronic history taking systems) exist. Only generalist publications are cited ([5–16], for review [5, 16]). Our approach has as starting point the fact that the collection of patient data can be considered as a patient data vector. Assignment of the symptoms or signs of a patient to a certain diagnosis is possible by a bundle of different not exclusive methods: (1) by the knowledge of the physician, (2) by comparison of the patient data with clinical pathways and guidelines, (3) by boards of specialized physicians, (4) a second opinion by an experienced medical doctor, and (5) by computer-assisted comparison with a data base of medical knowledge. The approach proposed in (5) implies, that we use formalized systems allowing sampling of medical knowledge (prototypic diseases) in a formalized manner. We describe thereafter a database of prototypes of diseases, which are oriented on the concept of vectors (patient vectors and disease vectors). For this approach three constituents are necessary: (1) patient vector, (2) a pool of disease vectors (described in this paper) (3) an algorithm allowing an interaction between both vectors. For the approach mentioned in (2) and (3) clinical decision support systems (CDSS and inference engine technology) are available [17–19]. Most approaches concerning computer-assisted data bases of medical knowledge are based on classical object-oriented structures and an entity-relationship-model. Substantial improvement of CDSS may be provided by IT-systems improving knowledge acquisition such as MetaMap [20] and cTake [21].

Our system shows that it is possible to use original atomistic language element and an intuitive, flexible semantic network to get medical context and structures which allow complex diagnostic understanding. The diagnosis process appears as a system driven dialogue which tries to match system semantics with patient semantics. This procedure can be understood as a “linguistic game”, which provides a “third opinion” for the patient’s symptoms.

## Methods

For the demonstration of our approach we used 4 examples of patients, fitting the symptoms of these patients to possible disease entities (pathosoms in our terminology).

### Examples

**Case 1**: The patient suffers from cytopenia, anemia and bone marrow: hypercellular (3 tuple, one with an attribute).

**Case 2**: A female, aged 55 years suffering from a firm lump in the mamma (2-tuple with 1 and 2 attributes).

**Case 3**: A newborn suffering from “polydactyly”, “renal cyst” and “encephalocele” (3-tuple).

**Case 4**: A female patient suffering from proteinuria, hematuria and fever (3-tuple).

### Thesauri

A thesaurus was defined as a collection of atomistic terms with a coding system (numeric, alpha-numeric or string). Many thesauri could be obtained from Internet or official institutions as the DIMDI (Deutsches Institut für medizinische Dokumentation) [22]. Examples of such medical thesauri are: ICD (International Classification of Diseases, injuries and causes of death [23], ICD-O (International Classification of Diseases, Oncology) [24], ORPHANET (Portal für seltene Krankheiten und Orphan Drugs) [25], TA (Taxonomy of Anatomy) [26], OPS (Operationen-und Prozedurenschlüssel -Internationale Klassifikation der Prozeduren in der Medizin 2015) [27], OMIM (Online Mendelian Inheritance in Man) [28], CAS (common chemistry data base) [29], EC (comprehensive enzyme information system) [30], HGNC (Hugo Gene Nomenclature Committee 2015) [31], ATC (Anatomical Therapeutic Chemical Classification) [32] and LOINC (Logical Observation identifiers and names) [33]. All these thesauri terms and codes are harmonized and included in Memem7 using our own coding system with links to the original code.

### Software

The system is focused on design flexibility using common website programming development environments like common SQL databases and Javascript.

### Atomistic approach

Each descriptor of a symptom or sign (pathological sign, laboratory sign, EKG report (electrocardiogram) or genetic findings are transformed to an atomistic term. Atomistic terms are the basic form of our system. An atomistic term can be considered as a stem word with a singular code and some added attributes. One example of such an atomistic term is proteinuria with a numeric attribute (g/ml) or headache with the attribute: strong. Attributes may be cardinal, ordinal or numeric.

### Prototypic diseases (pathosom)

A disease entity or a prototype of a disease is considered as the sum of all descriptors in a WHO classification or clinical pathway. All these descriptors must be atomic (see below for definition of a pathophem). Pictures are, so far, included by internet links. All these descriptors were class-divided as shown in Table 1. A pathosom is therefore defined as the sum of a vector: c (j1…jn). Each element j can be assigned to certain classes (see Table 1) or thesaurus (see Table 2).

**Table 1:** Classes of Memem7

No	Class	Subclass	Subclass2	Elements
1100	Description	Definition		7241
1400	Description	System/Lokalisation		9875
1500	Description	Struktur	ElementOf	1733
1540	Description	Struktur	HasElement	6251
1550	Description	Struktur	HasVariante	1651
1570	Description	Struktur	Sekundare Form	177
5100	Symptoms	Anamnese		7406
5110	Symptoms	Anamnese	Akut	182
5130	Symptoms	Anamnese	Vorgeschichte	479
5140	Symptoms	Anamnese	Familie	47
5150	Symptoms	Anamnese	Demografisch	10
5160	Symptoms	Anamnese	Sozial	88
5300	Symptoms	Vital		7597
5400	Symptoms	Physikal		768
5410	Symptoms	Physikal	Spirometry	22
5500	Symptoms	Labor		4423
5510	Symptoms	Labor	Clinical	696
5520	Symptoms	Labor	Agent	1370
5530	Symptoms	Labor	Toxicology	35
5600	Symptoms	Imaging		401
5610	Symptoms	Imaging	Ultrasound	119
5620	Symptoms	Imaging	Radiology	570
5630	Symptoms	Imaging	MRT	187
5640	Symptoms	Imaging	CT	132
5650	Symptoms	Imaging	Endoskopie	16
5700	Symptoms	Pathologie		1795
5710	Symptoms	Pathologie	Makroskopie	1252
5720	Symptoms	Pathologie	Mikroskopie	8576
5725	Symptoms	Pathologie	Elektronenmikroskopie	291
5730	Symptoms	Pathologie	Spezialfarbung	236
5735	Symptoms	Pathologie	Enzymhistochemie	96
5738	Symptoms	Pathologie	Zytologie	894
5740	Symptoms	Pathologie	Immunhistochemie	3596
5745	Symptoms	Pathologie	FACS	29
5750	Symptoms	Pathologie	In-Situ Hybridisierung	39
5760	Symptoms	Pathologie	Molekularbiologie	30
5770	Symptoms	Pathologie	Stoffe	95
5795	Symptoms	Pathologie	Differenzialdiagnose	1356
5800	Symptoms	Genetik		4400
5900	Symptoms	Psychologie		175
6100	Characteristics	Historie		83
6300	Characteristics	Epidemologie		2029
6310	Characteristics	Epidemologie	Sex	557
6320	Characteristics	Epidemologie	Age	989
6330	Characteristics	Epidemologie	Race	66
6340	Characteristics	Epidemologie	Region	132
6350	Characteristics	Epidemologie	Inzidenz	198
6360	Characteristics	Epidemologie	Pravalenz	528
6400	Characteristics	Atiologie		607
6500	Characteristics	Pathophysiologie		2783
6600	Characteristics	Verlauf		588
6610	Characteristics	Verlauf	Beginn	236
6620	Characteristics	Verlauf	Verlauf	266
6630	Characteristics	Verlauf	Stadium	462
6640	Characteristics	Verlauf	Prognose	1122
6650	Characteristics	Verlauf	Komplikation	1506
6660	Characteristics	Verlauf	Risikofaktor	965
6700	Characteristics	Komorbiditat		1316
6800	Characteristics	Differentialdiagnose		7785
6900	Characteristics	Untersuchung		3016
8100	Therapy	Therapieprinzipien		1422
8200	Therapy	Medikamente		2302
8300	Therapy	Chirurgie		493
8400	Therapy	Strahlentherapie		32
8500	Therapy	Ambulance		8
8600	Therapy	ReHa		44
8700	Therapy	Psychotherapie		7
8800	Therapy	Alternative		369
9800	Therapy	Vorsorge		326

**Table 2:** Thesauri in medical use [21–29, 35, 36, 38]

Thesaurus (acronym)	Content of diseases	Free available and used in memem7
ICD-10-GM	Diseases	yes
ICD-O	Morphology of tumors	yes
ORPHANET	Rare Diseases	yes
OPS	Medical interventions	yes
LOINC	Laboratory Codings	yes
SNOMED	Medical terms and ontology	no
OMIM	Human genes/Genetic Disorders	yes
TA	Taxanomica anatomica	yes
CAS	Molecules	yes
EC	Enzymes	yes
HGNC	Molecular biological terms	yes
ATC	Agents/Medicaments	yes

### Pathophem

A pathophem is an object sentence in a classification, clinical pathway or a publication after its atomistic representation. After this breakdown, we have an object (a single word meaning a stem word in the linguistic sense) which can be further characterized by up to three attributes or any object. Attributes are time, intensity, color, taste etc. Each object can be either true or false or not available. Objects should be coded by the thesaurus systems as given in Table 2. All content is coded along our coding system and then termed pathophem. In addition, occurrence probabilities between 0 and 1 can be considered. “Lead” means that this pathophem is always present in a pathosom and signifies a probability of 1, “+++” means a frequency between 50% to 99.9% or a probability of 0.5–0.99, “++” means a frequency of 10% to 49.9% or a probability of 0,1–0.49, “+” 1% to 9.9% or a probability of 0.01–0.09, and “(+)” <1% or a probability <0.01. “NOT” means that a pathophem excludes a pathosom.

### Diagnostic algorithm (linguistic game)

The algorithm uses various syntactical and semantic analysis procedures to provide a set of diagnoses. Proposal of diagnoses occurs by matching a patient vector (medical report replaced in our test system by a 5-tuple vector of patient data) with the disease vectors (termed pathosoms). First the patient vector must be analyzed syntactically. This verification ensures that all terms are within the basic thesauri and have a “primary meaning” within the semantic network. Our semantic network is a classical semantic network for knowledge representation. It is a directed graph consisting of vertices, which represent terms, objects and concepts, and edges, which represent semantic relations between terms like “is-a”, “is-part-of”, “has-attribut-of”, “is-class-of”.

There will be a continuous dialog by offering alternative meanings if all terms are completely matched using synonyms, clarifying double meanings etc. The first result will be a set of matching pathosoms with a ranking. If the set is too large the system provides a second analysis to reduce the set by asking more details about other essential symptoms of the pathosoms. If the set is very small or empty the system tries to broaden the patient vector by using semantic network context methods like “is-class” and others. For example: If you are searching for a concrete finger symptom/pathophem and you cannot find anyone there might be a corresponding symptom/pathophem about the hand which may fit.

### Linguistic problems (German/English)

More than 85% of the used terms are already translated in English.

### Statistical methods

For calculations, we used R statistic package (version 3.1.1). For analysis of the ICD-10 (German version) we used the tm package of R [34]. The question to be answered was of how many atomized terms the ICD-10 consisted of and to integrate those lacking in memem7.

### Description of software

- Project name: Memem7 Medical Semantic Network
- Project home page: not yet determined
- Archived version: not yet determined
- Operating system(s): Platform independent
- Programming language: Coldfusion, Javascript, HTML5
- Other requirements: Coldfusion Server
- License: Not yet determined
- Any restrictions to use by non-academics: Not yet determined

### Evaluation of Memem7

Testing the quality of Memem7 was done with 190 artificial reports (simulated cases not representing a patient), originally developed for testing CLEOS [3–5]. Each artificial case consists of a short medical report and/or symptoms with a mono-causal medical explanation.

## Results

### Examples for using Memem7 for searching

This means comparing the patient vector (a tuple with up to 5 elements) with the set of pathosoms, looking for those pathosoms which fits best to the patient vector using a linguistic approach.

### Case 1

The search terms in English are cytopenia (41), anemia (657), bone marrow (91): hypercellular. This is a patient vector with 3 elements. The numbers in parenthesis signify how often a pathophem is mentioned in Memem7. For the intersection of the three pathophems only one pathosom was found, namely RAEB (M3501, see Additional file 1).

### Case 2

The search terms are female (597): aged 55 years, nodule (365): firm (90), nodule: mamma (434). The pathosom invasive breast cancer was found and (excluding the attribute firm) 6 additional pathosoms are proposed: angiosarcoma of breast, secretory breast cancer, benign breast tumor, pseudoangiomatoid stroma hyperplasia, fibroadenoma and breast papilloma were recognized.

### Case 3

The search terms are polydactyly (40), renal cyst (14), encephalocele (6) in a newborn infant. 3 pathosoms fitted to the search terms: Meckel syndrome type 1,3,6.

### Case 4

The search terms are being proteinuria (61) and hematuria (72). As a result, 25 pathosoms are given by Memem7 as possible disease vectors fitting both symptoms. Adding fever (622) to the atomistic terms gives 8 additional pathosoms: cryoglobulinemia with vasculitis, periarteriitis nodosa, microscopic polyangiitis, hanta virus infection, hemorrhagic fever with with renal syndrome, emphysematous pyelonephritis, infective glomerulonephritis and acute interstitial nephritis.

### Examples of pathosoms fitting the cases 1–4

#### Case 1: RAEB (refractory anemia with excess of blasts)

As an example, we have chosen the disease entity of RAEB being a subset of myelodysplasia. This entity is described by two pages (pp100–101, 22] in the WHO classification of tumors of hematopoietic and lymphoid tissue [35]. The ICD-10-GM coding is D46.2. The ICD-O-coding 9983/3. The first sentence is: refractory anemia with excess blasts is a myelodysplastic syndrome (MDS) with 5–19% myeloblasts in the bone marrow (BM) or 2–19% blasts in the peripheral blood. In our system, this sentence is transformed in a vector with atomistic terms: c (refractory anemia, myeloblasts: bone marrow > 5%: AND: myeloblasts: bone marrow < 20%) OR (blasts: peripheral blood > 2%). The whole Boole term must be TRUE. This is an example of generating pathophemes in the pathosom RAEB. The coded version of this sentence is: M3094|; (O812| & 10209|: 5..20%) | (10221 T| &10209 T|: 2..20%). For details see also Additional file 1: Tables S1 to S3.

#### Case 2: invasive breast cancer

Invasive breast cancer is a set of clinical and morphological diseases with a hierachical structure. There are 68 pathosoms of breast cancer including terms like non-invasive breast cancer, inflammatory breast cancer (see Additional file 1: Tables S5 and S6) or tubular carcinoma of breast, which are structured in a tree (“is-element”, “has-element”, “has-variant”). One example of these 68 pathosoms (inflammatory breast cancer) is given in Table 3 and Additional file 1: Tables S5 and S6.

**Table 3:**
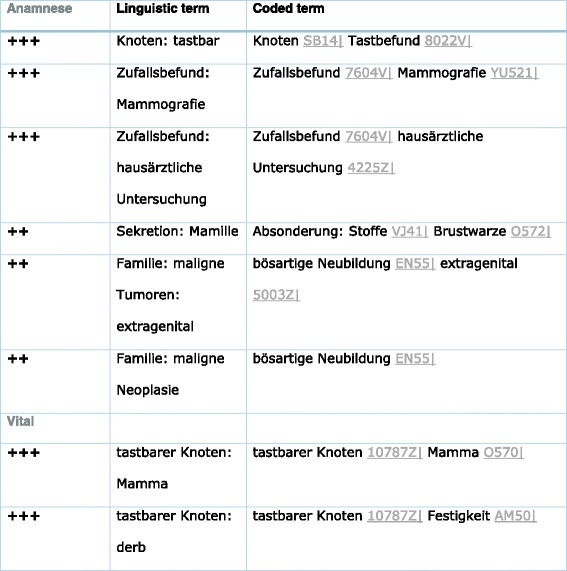
Part of the pathosom breast cancer with concern to anamnesis and examination by the physican (Vital)

Note that “tastbarer Knoten is coded by 10787Z and this code includes lump and palpable node

#### Case 3: genetic disorder of skeletal disease

For this example, we used the Meckel syndrome type IV (see Table 4) [36, 37]. The atomistic terms are coded. If different codes are available from different thesauri the selection of the assignment is random. As shown in Table 1, 6 different synonyms or codes (such as the OMIM code 612284) are provided in our system allowing to collect further information.

**Table 4:**
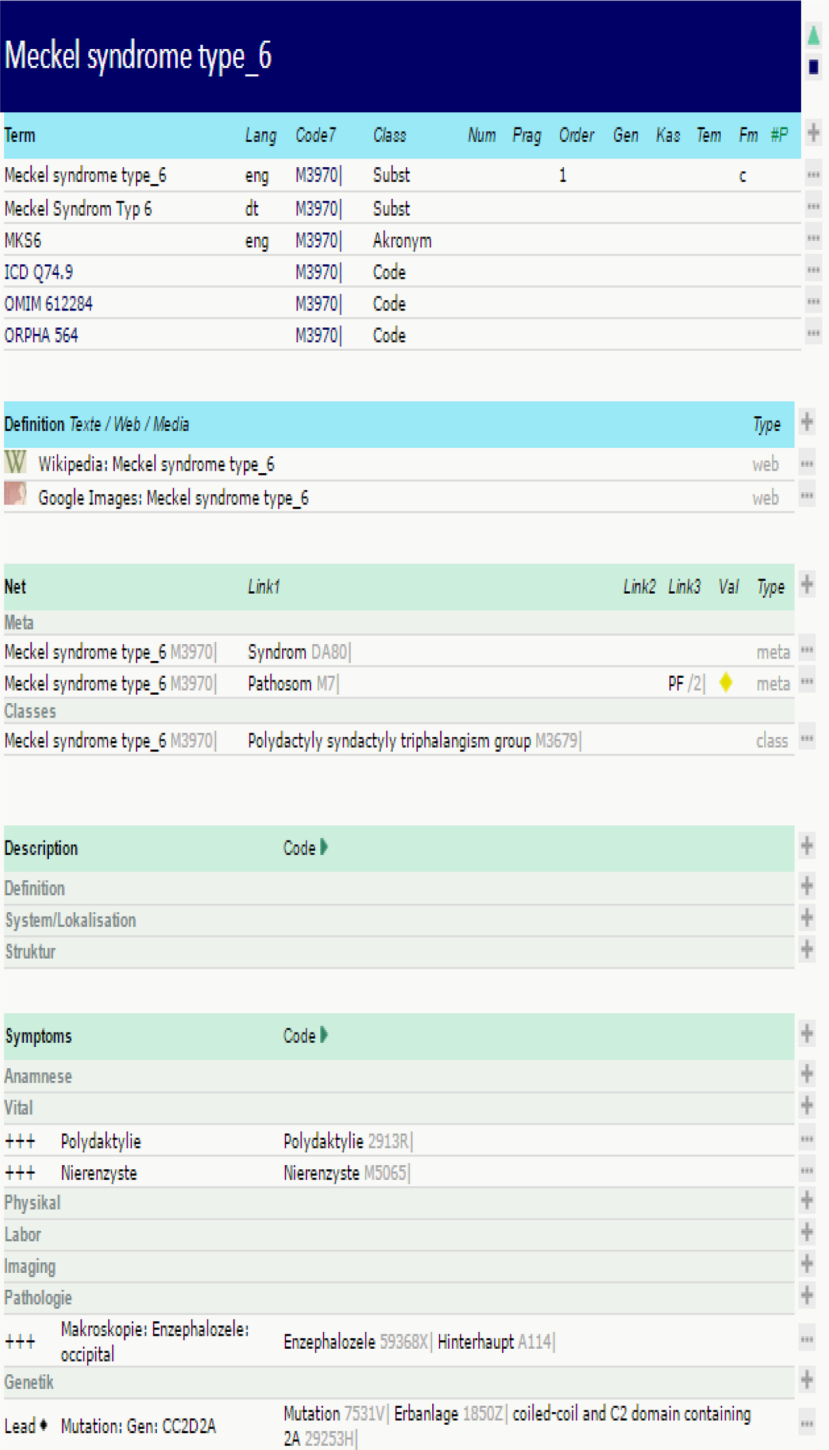
Meckel syndrom VI as an example of a pathosom

#### Case 4: membrano-proliferative glomerulonephritis (MPGN) (see Table 5)

Classification of MPGN is a set of three subsets termed MPGN Type I, MPGN type II and MPGN type III [38]. The ICD coding is N05.5, N04.5, N03.5, N02.5, and N01.5 (see Additional file 1: Tables S7 and S8).

**Table 5:**
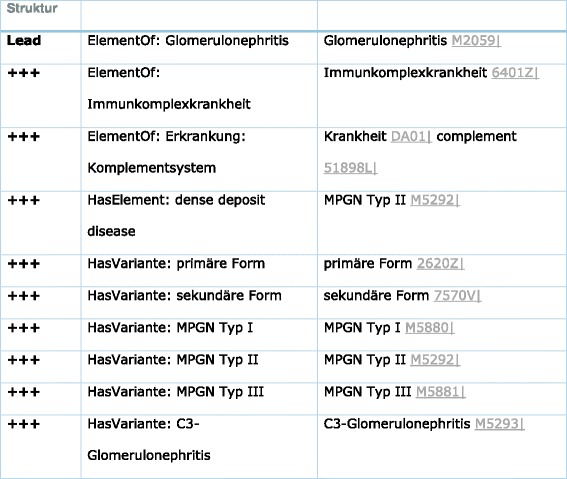
Note that each pathosom is part of a hierarchical tree with superset and subsets

Note that syntactic differences or language problems are not of interest as “Nierenzyste”, “Nierencyste”, “renal cyst” and “renal cysts” are all coded by M5065

### Description of Memem7

#### Thesauri

The thesauri used in our system are listed in Table 2. For the example RAEB (refractory anemia with excess of blasts) we have one alpha-numeric code ICD-10 D46.2. In the other thesauri RAEB is not mentioned. In the ICD-O classification [23], however, this disease entity is mentioned by the term refractory cytopenia with multilineage dysplasia. For the second example with genetic caused skeletal disorder different ICD-10 codes are available (ICD-10 Q65.–Q79.9). With the tm text analysis system of R, we obtained a list of 53947 atomized terms. 16798 (31.1%) of these ICD-10 terms were originally not part of memem7 and were integrated in the system. Mallet finger (Hammerfinger in German) is part of ICD-10 (M20.0), but originally not of Memem7. Tm identifies the term Hammerfinger as element of ICD-10 and this term was added to Memem7 (including its counterpart in English). The number of diagnoses in the ICD-10 code (not signs, symptoms) was estimated to ~ 9700.

### Number of pathosoms and pathophemes

By now approx. 4600 pathosoms (prototypic diseases) with 104.200 pathophemes (prototypic symptoms) are included in our system. For writing the atomized pathophems we provide terms taken from different thesauri (see Table 2). In total, there are approx. 230.000 terms coded out of 1.550.000 terms including German, English and Latin in different grammatical forms. Therefore, Memem7 covers ~ 4600/9700 (47.2%) of the diagnoses of the ICD-10 classification system. We assume that each pathosom consists of at least 10…100 pathophemes depending of the complexity of the disease.

### Comparison of pathosom and diagnosis

A pathosom is a data model which describes knowledge about a disease in a structured but flexible, non-deterministic way. It also may be considered as a vector of pathophems, each of them being TRUE (1), FALSE (0), or by an evidence between 0 and 1. A diagnosis is either an alpha-numeric code such as a ICD-10 code [23, 24, 35] or a written description of a disease such as refractory anemia with an excess of blasts (RAEB) [35]. RAEB exists in three variants, termed RAEB-1, RAEB-2 and RAEB-F, being subsets of the RAEB pathosom. A pathosom is a vector of pathophems. RAEB is a vector with 75 elements (so far) (see Additional file 1: Tables S1 to S4) with three variants. Each pathosom is part of a tree structure. RAEB has three subsets: RAEB-1, RAEB-2 and RAEB-F and is an element of the pathosom myelodysplasia. The tree structure is one of the basic structure for the semantic network and provides “has-attribute” relationships.

### Evaluation of the system

In 90/190 (47.4%) artificial cases the proposed diagnosis was identified by memem7 (47.6%). The number of proposed pathosoms (diagnoses) ranged between 0 and 173 with a median of 3.

## Discussion

Nowadays, it is common sense that both computational linguistics and computer technology will change the practice of medicine fundamentally [39]. However, the evidence, that this change the quality of medicine will improve is sparse [18].

Here, we describe a software system consisting of a pool of pathosoms (disease entities, syndromes, symptoms or sets of disease entities). The goal of this publication is to demonstrate that with some prerequisites a computer-assisted assignment of symptoms (in our preliminary system a 5n tuple) to certain disease entities is possible. The prerequisites are (1) linguistic knowledge is delivered atomistic (2) each pathosom (prototypic disease entity) is considered as a vector (3), and the results of the analysis should be a linguistic game assisting classification of a patient vector to different pathosoms without the use of defined mathematical methods or a distance measure. After the search function of Memem7 a list of possible pathosoms explaining the patient’s complaint is given, so far, with focus on linguistic and semantic network heuristics.

For a coming assistance system of decision making in medicine three different tools are mandatory: (1) an electronic history taking which can be transformed in a patient data vector [3–5] or extracts of electronic health care systems (2) a set of disease vectors (in our system called pathosoms) each reflecting a prototypic disease vector or an algorithms for a disease entity. (3) an algorithm comparing the patient vector with all pathosom vectors giving a ranking of possible pathosoms fitting to a given patient vector (inference engine).

Our system termed Memem7 allows today: (1) to construct a prototypic disease vector (2) to have a restricted dialogue with a 5n-tuple of a patient vector. Restricted dialogue means that, so far, only 5 tuples can be analyzed by Memem7. After this dialogue, the user is provided with n pathosoms, which fit to the 5-tuple patient vector. The goal of our approach was to demonstrate the feasibility of a knowledge collection in a formalized way.

The mathematical methods used in big data analysis will be of further interest for improving the tools allowing machine assisted medical treatment decision as described by Sun and coworkers, Slack and coworkers and Huang and coworkers [40–42]. Our system covers approximately 4600 medical diagnoses (increasing constantly). Many trials of electronic history taking, even in very sophisticated technical settings as described by Morrison and coworkers [43] are published (70 up to 2010) ([3, 4], for review [5, 16]). However, so far, as the authors survey the literature only few open source application of an electronic history taking system are available such as OpenEMR and OpenEHR [44, 45]. In our linguistic game these histories taking systems were replaced by a 5n-tupel. The term linguistic game has some fundamental implications (1) error is part of any game (2) decision can be assisted, but not replaced by a linguistic game (3) a player can improve his results by improving his knowledge (in our system the disease vector of the pathosoms). Therefore, our system may be considered as a third opinion to a given patient vector with so far 5 tuples. Combining memem7 with tools recognizing medical texts like MetaMap [20] or cTake [21] are currently in work.

Can we give any data for accuracy of memem7? In a first testing of the system with up to 5 tuple of search terms we identified the proposed diagnosis of 190 artificial cases for 46.7%. The data available for assessment of the sensitivity for clinical decision support system (CDSS) are sparse and difficult to compare. In one recent publication of Müller and coworkers [46] a sensitivity of 96% was claimed, when case reports of the New England Journal of Medicine were used for the evaluation of the systems (DXplain, Isabel Healthcare, Diagnosis Pro, PEPID) [47–50]. Our much less impressive data will improve with each new test as the mistakes found in the test are corrected after completing the test. Therefore, the tests are part of a continuous improvement quality circle which provides algorithms to enhance both sematic network and pathophemes (disease symptoms).

What are the differences of Memem7 to other existing medical decisions systems like e.g. HELP [18]. There are different approaches for use of computer-technology in CDSS such as probabilistic, Bayesian approaches or machine learning systems. We add a system based on atomized linguistic terms. It may be possible to combine probabilistic and linguistic approaches. The advantage of a linguistic system is that atomized terms (pathophems) can be easily added to a disease entity. The comparison between a linguistic and a probabilistic approach or its combined use is planned on basis of the next software version.

There are already many systems and tools presented for biomedical text mining and biomedical concept recognition [51, 52]. The systems mainly focus on natural language processing and concept identification through dictionary matching and various machine learning mechanisms. In contrast to these approaches Memem7 is not focused on identifying concepts in natural languages but rather on using such concepts for identification of other complex objects like health disorders. In future, the system might also be able to handle the pre-process of concept identification of written data e.g. medical patient reports. Technically Memem7 is dealing with the problem of matching ambiguous fact concepts (like symptoms, medical events, lab data) with complex concept trees (like anatomy, medical body functions, diseases). Because of the plurivalent nature of the concepts and the heuristic of the algorithms the matching processing is more like a game than a deterministic process.

## Conclusions

In conclusion, we present an electronic system of disease entities, which can enter in a formalized dialog with a patient data vector as proof of concept of a medical navigation system based on a linguistic game.

## Additional file

Additional file 1: Table S1. RAEB part 1. **Table S2.** RAEB part 2. **Table S3.** RAEB part 3. **Table S4.** RAEB part 4. Note that the underscored pathophem (IPSS Score) is not yet included in the system. **Table S5.** Inflammatory breast cancer part 1. **Table S6.** Inflammatory cancer part 2. **Table S7.** Membrano-proliferative glomerulonephritis part 1. **Table S8.** membrano-proliferative glomerulonephritis part 2. (DOCX 174 kb)

## Abbreviations

ATC: Anatomical therapeutic chemical classification
CAS: Common chemistry data base
CDSS: Clinical decision support system
DIMDI: Deutsches Institut für medizinische Dokumentation
EC: Brenda, comprehensive enzyme information system
HGNC: Hugo gene nomenclature committee 2015)
HTML5: Hypertext markup language
ICD-10: International classification of diseases, injuries and causes of death
ICD-O: International classification of diseases, oncology
LOINC: Logical observation identifiers and names
MPNG: Membrano-proliferative glomerulonephritis
OMIM: Online Mendelian Inheritance in Man
OPS: Operationen-und Prozedurenschlüssel-Internationale Klassifikation der Prozeduren in der Medizin 2015
ORPHANET: Portal für seltene Krankheiten und Orphan Drugs
RAEB: Refractory anaemia with excess blasts
TA: Taxonomy of anatomy
WHO: World Health Organization
