# Supplementary material for: Medical diagnosis as a linguistic game
Source: BMC Med Inform Decis Mak. 2017 Jul 10;17:103. doi: 10.1186/s12911-017-0488-3 (PMC5504712; doi:10.1186/s12911-017-0488-3)
Supplement: Additional file 1: Table S1. — RAEB part 1. Table S2. RAEB part 2. Table S3. RAEB part 3. Table S4. RAEB part 4. Note that the underscored pathophem (IPSS Score) is not yet included in the system. Table S5. Inflammatory breast cancer part 1. Table S6. Inflammatory cancer part 2. Table S7. Membrano-proliferative glomerulonephritis part 1. Table S8. membrano-proliferative glomerulonephritis part 2. (DOCX 174 kb) [file 12911_2017_488_MOESM1_ESM.docx]

| Refractory anaemia with excess blasts | | | | | | | | | | | | | [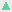](http://85.214.20.219/memem7/Terms_List.cfm?modus=&search=M3101\|&task=tree&refresh=1)  [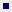](http://85.214.20.219/memem7/Terms_List.cfm?modus=&search=M3101\|&task=syn&refresh=1) |  |
| --- | --- | --- | --- | --- | --- | --- | --- | --- | --- | --- | --- | --- | --- | --- |
| **Term** | *Lang* | *Code7* |  | *Class* | *Num* | *Prag* | *Order* | *Gen* | *Kas* | *Tem* | *Fm* | *#P* | [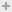](http://85.214.20.219/memem7/Terms_NewUpdate.cfm?code7=M3101\|) | Formularbeginn  Formularende |
| Refractory anaemia with excess blasts | eng | [M3101\|](http://85.214.20.219/memem7/Terms_List7.cfm?search=M3101\|) |  | Subst |  |  | 1 |  |  |  | c | [5](http://85.214.20.219/memem7/TermsPhemes_List.cfm?link7=M3101\|&concept=100C) | [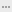](http://85.214.20.219/memem7/Terms_Edit.cfm?txt=Refractory%20anaemia%20with%20excess%20blasts&lang=eng&code7=M3101\|&class=S) |  |
| Refraktäre Anämie mit Blastenüberschuss | dt | [M3101\|](http://85.214.20.219/memem7/Terms_List7.cfm?search=M3101\|) |  | Subst |  |  |  |  |  |  | c |  | [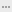](http://85.214.20.219/memem7/Terms_Edit.cfm?txt=Refraktäre%20Anämie%20mit%20Blastenüberschuss&lang=&code7=M3101\|&class=S) |  |
| RAEB | eng | [M3101\|](http://85.214.20.219/memem7/Terms_List7.cfm?search=M3101\|) |  | Akronym |  |  |  |  |  |  |  |  | [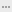](http://85.214.20.219/memem7/Terms_Edit.cfm?txt=RAEB&lang=eng&code7=M3101\|&class=K) |  |
| [ICD D46.2](http://85.214.20.219/memem7/ICDX_List.cfm?icx=D46.2) |  | [M3101\|](http://85.214.20.219/memem7/Terms_List7.cfm?search=M3101\|) |  | Code |  | | | | | | | | [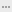](http://85.214.20.219/memem7/TermsCodes_Edit.cfm?txt=D46.2&lang=ICD&code7=M3101\|&class=C) |  |
| [ICD-O M9983/3](http://85.214.20.219/memem7/ICO_List.cfm?search=M9983/3) |  | [M3101\|](http://85.214.20.219/memem7/Terms_List7.cfm?search=M3101\|) |  | Code |  | | | | | | | | [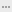](http://85.214.20.219/memem7/TermsCodes_Edit.cfm?txt=M9983/3&lang=ICO&code7=M3101\|&class=C) |  |

|  | | | | |  |  |
| --- | --- | --- | --- | --- | --- | --- |
|  |  |  |  |  |  |  |
| **Definition** *Texte / Web / Media* | | *Type* | | | [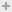](http://85.214.20.219/memem7/TermsDef_NewUpdate.cfm?code7=M3101\|) |  |
| [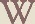](http://flexikon.doccheck.com/de/Refraktäre%20Anämie) | DocCheck: Als refraktäre Anämie bezeichnet man eine Form des myelodysplastischen [Syndroms\|](http://85.214.20.219/memem7/Terms_List.cfm?search=Syndroms\|), dessen Ursache auf eine gestörte [Proliferation\|](http://85.214.20.219/memem7/Terms_List.cfm?search=Proliferation\|) und [pathologische\|](http://85.214.20.219/memem7/Terms_List.cfm?search=pathologische\|), sowie [morphologische\|](http://85.214.20.219/memem7/Terms_List.cfm?search=morphologische\|) Veränderung der [Erythroblasten\|](http://85.214.20.219/memem7/Terms_List.cfm?search=Erythroblasten\|) zurückzuführen ist. | web |  |  | [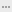](http://85.214.20.219/memem7/TermsDef_Edit.cfm?ddid=10189) |  |
| [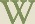](https://de.wikipedia.org/wiki/Refraktäre%20Anämie%20mit%20Blastenüberschuss) | Wikipedia: Refraktäre Anämie mit Blastenüberschuss | web |  |  | [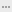](http://85.214.20.219/memem7/TermsDef_Edit.cfm?ddid=10190) |  |
| [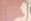](https://www.google.de/search?site=imghp&tbm=isch&source=hp&q=Refraktäre%20Anämie%20mit%20Blastenüberschuss) | Google Images: Refraktäre Anämie mit Blastenüberschuss | web |  |  | [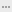](http://85.214.20.219/memem7/TermsDef_Edit.cfm?ddid=10191) |  |
|  | | | | |  |  |

|  | | | | | | | | | | |  |  |
| --- | --- | --- | --- | --- | --- | --- | --- | --- | --- | --- | --- | --- |
| **Net** |  | *Link1* | *Link2* | *Link3* | *Val* | | | | *Type* | | [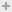](http://85.214.20.219/memem7/TermsLinks_NewUpdate.cfm?code7=M3101\|) |  |
| **Meta** | | | | | | | | | | |  |  |
| Refractory anaemia with excess blasts [M3101\|](http://85.214.20.219/memem7/Terms_List7.cfm?search=M3101\|) |  | Krankheit[DA01\|](http://85.214.20.219/memem7/Terms_List7.cfm?search=DA01\|) |  |  |  |  |  |  | meta |  | [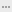](http://85.214.20.219/memem7/TermsLinks_Edit.cfm?llid=135259&code7=M3101\|) |  |
| Refractory anaemia with excess blasts [M3101\|](http://85.214.20.219/memem7/Terms_List7.cfm?search=M3101\|) |  | Pathosom[M7\|](http://85.214.20.219/memem7/Terms_List7.cfm?search=M7\|) |  | PF[/2\|](http://85.214.20.219/memem7/Terms_List7.cfm?search=/2\|) | [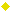](http://85.214.20.219/memem7/TermsLinks_StatusUpdate.cfm?code7=M3101\|&llid=6554&status=3) |  |  |  | meta |  | [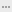](http://85.214.20.219/memem7/TermsLinks_Edit.cfm?llid=6554&code7=M3101\|) |  |
| **Classes** | | | | | | | | | | |  |  |
| Refractory anaemia with excess blasts [M3101\|](http://85.214.20.219/memem7/Terms_List7.cfm?search=M3101\|) |  | Myelodysplastische Syndrome[998N\|](http://85.214.20.219/memem7/Terms_List7.cfm?search=998N\|) |  |  |  |  |  |  | class |  | [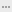](http://85.214.20.219/memem7/TermsLinks_Edit.cfm?llid=48771&code7=M3101\|) |  |
| Refractory anaemia with excess blasts [M3101\|](http://85.214.20.219/memem7/Terms_List7.cfm?search=M3101\|) |  | Neoplasie[EN50\|](http://85.214.20.219/memem7/Terms_List7.cfm?search=EN50\|) |  |  |  |  |  |  | class |  | [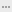](http://85.214.20.219/memem7/TermsLinks_Edit.cfm?llid=144271&code7=M3101\|) |  |

|  | | | | | | | | | | |  |  |
| --- | --- | --- | --- | --- | --- | --- | --- | --- | --- | --- | --- | --- |
|  |  |  |  |  |  |  |  |  |  |  |  |  |
| **Description** | | | | |  | [Code[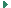](http://85.214.20.219/memem7/TermsPhemes_TransUpdate.cfm?code7=M3101\|&cl=1&concept=100C)](http://85.214.20.219/memem7/TermsPhemes_TransUpdate.cfm?code7=M3101\|&cl=1&concept=100C) |  |  |  |  |  |  |
| **Definition** | | | | | | | | | | | [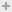](http://85.214.20.219/memem7/TermsPhemes_New.cfm?code7=M3101\|&cl=11&concept=100C) |  |
| +++ |  |  |  | Myelodysplasie: Knochenmark: Myeloblasten 5:19 %\| |  | Myelodysplasie [M1330\|](http://85.214.20.219/memem7/Terms_List7.cfm?search=M1330\|) Knochenmark [O812\|](http://85.214.20.219/memem7/Terms_List7.cfm?search=O812\|) Myeloblast [10209T\|](http://85.214.20.219/memem7/Terms_List7.cfm?search=10209T\|) |  |  |  |  | [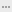](http://85.214.20.219/memem7/TermsPhemes_Edit.cfm?ppid=81264&concept=100C) |  |
| +++ |  |  |  | Myelodysplasie: Blutausstrich: Myeloblasten 2:19 %\| |  | Myelodysplasie [M1330\|](http://85.214.20.219/memem7/Terms_List7.cfm?search=M1330\|) Ausstrich peripheres Blut [10221T\|](http://85.214.20.219/memem7/Terms_List7.cfm?search=10221T\|) Myeloblast [10209T\|](http://85.214.20.219/memem7/Terms_List7.cfm?search=10209T\|) |  |  |  |  | [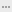](http://85.214.20.219/memem7/TermsPhemes_Edit.cfm?ppid=81265&concept=100C) |  |
| +++ |  |  |  | Myelodysplasie: Myeloblasten: AND: Auer Stäbchen |  | Myelodysplasie [M1330\|](http://85.214.20.219/memem7/Terms_List7.cfm?search=M1330\|) Myeloblast [10209T\|](http://85.214.20.219/memem7/Terms_List7.cfm?search=10209T\|) AND [.AND\|](http://85.214.20.219/memem7/Terms_List7.cfm?search=.AND\|) Auerstäbchen [10310T\|](http://85.214.20.219/memem7/Terms_List7.cfm?search=10310T\|) [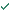](http://85.214.20.219/memem7/TermsPhemes_FixUpdate.cfm?ppid=81266&code7=M3101\|) |  |  |  |  | [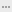](http://85.214.20.219/memem7/TermsPhemes_Edit.cfm?ppid=81266&concept=100C) |  |
| **System/Lokalisation** | | | | |  |  |  |  |  |  | [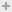](http://85.214.20.219/memem7/TermsPhemes_New.cfm?code7=M3101\|&cl=14&concept=100C) |  |
| Lead | 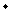 |  |  | Knochenmark |  | Knochenmark [O812\|](http://85.214.20.219/memem7/Terms_List7.cfm?search=O812\|) |  |  |  |  | [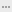](http://85.214.20.219/memem7/TermsPhemes_Edit.cfm?ppid=81267&concept=100C) |  |
| Lead | 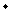 |  |  | peripheres Blut |  | Ausstrich peripheres Blut [10221T\|](http://85.214.20.219/memem7/Terms_List7.cfm?search=10221T\|) |  |  |  |  | [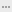](http://85.214.20.219/memem7/TermsPhemes_Edit.cfm?ppid=81268&concept=100C) |  |
| **Struktur** | | | | |  |  |  |  |  |  | [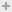](http://85.214.20.219/memem7/TermsPhemes_New.cfm?code7=M3101\|&cl=15&concept=100C) |  |
| +++ |  |  |  | HasElement: RAEB-1 |  | RAEB-1 [M3110\|](http://85.214.20.219/memem7/Terms_List7.cfm?search=M3110\|) |  |  |  |  | [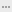](http://85.214.20.219/memem7/TermsPhemes_Edit.cfm?ppid=81262&concept=100C) |  |
| +++ |  |  |  | HasElement: RAEB-2 |  | RAEB-2 [M3112\|](http://85.214.20.219/memem7/Terms_List7.cfm?search=M3112\|) |  |  |  |  | [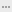](http://85.214.20.219/memem7/TermsPhemes_Edit.cfm?ppid=81263&concept=100C) |  |
| +++ |  |  |  | HasElement: RAEB-F |  | RAEB-F [M3111\|](http://85.214.20.219/memem7/Terms_List7.cfm?search=M3111\|) |  |  |  |  | [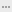](http://85.214.20.219/memem7/TermsPhemes_Edit.cfm?ppid=81300&concept=100C) |  |
|  |  |  |  |  |  |  |  |  |  |  |  |  |
| **Symptoms** | | | | |  | [Code[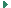](http://85.214.20.219/memem7/TermsPhemes_TransUpdate.cfm?code7=M3101\|&cl=5&concept=100C)](http://85.214.20.219/memem7/TermsPhemes_TransUpdate.cfm?code7=M3101\|&cl=5&concept=100C) |  |  |  |  |  |  |
| **Anamnese** | | | | | | | | | | | [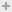](http://85.214.20.219/memem7/TermsPhemes_New.cfm?code7=M3101\|&cl=51&concept=100C) |  |
| +++ |  |  |  | Fieber |  | Fieber [EF\|](http://85.214.20.219/memem7/Terms_List7.cfm?search=EF\|) |  |  |  |  | [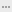](http://85.214.20.219/memem7/TermsPhemes_Edit.cfm?ppid=92280&concept=100C) |  |
| +++ |  |  |  | Abgeschlagenheit |  | Abgeschlagenheit [EB43\|](http://85.214.20.219/memem7/Terms_List7.cfm?search=EB43\|) |  |  |  |  | [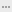](http://85.214.20.219/memem7/TermsPhemes_Edit.cfm?ppid=92281&concept=100C) |  |
| +++ |  |  |  | Infektanfälligkeit |  | Infektanfälligkeit [M1532\|](http://85.214.20.219/memem7/Terms_List7.cfm?search=M1532\|) |  |  |  |  | [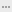](http://85.214.20.219/memem7/TermsPhemes_Edit.cfm?ppid=92282&concept=100C) |  |
| **Vital** | | | | |  |  |  |  |  |  | [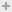](http://85.214.20.219/memem7/TermsPhemes_New.cfm?code7=M3101\|&cl=53&concept=100C) |  |

Additional File 1: Table S1. RAEB part 1

| **Physikal** | | | | |  |  |  |  |  |  | [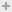](http://85.214.20.219/memem7/TermsPhemes_New.cfm?code7=M3101\|&cl=54&concept=100C) |  |
| --- | --- | --- | --- | --- | --- | --- | --- | --- | --- | --- | --- | --- |
| **Labor** | | | | |  |  |  |  |  |  | [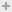](http://85.214.20.219/memem7/TermsPhemes_New.cfm?code7=M3101\|&cl=55&concept=100C) |  |
| +++ |  |  |  | Anämie |  | Anämie [M2024\|](http://85.214.20.219/memem7/Terms_List7.cfm?search=M2024\|) |  |  |  |  | [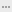](http://85.214.20.219/memem7/TermsPhemes_Edit.cfm?ppid=81271&concept=100C) |  |
| +++ |  |  |  | Neutropenie |  | erniedrigte Anzahl von Leukozyten [ML915\|](http://85.214.20.219/memem7/Terms_List7.cfm?search=ML915\|) |  |  |  |  | [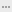](http://85.214.20.219/memem7/TermsPhemes_Edit.cfm?ppid=81272&concept=100C) |  |
| +++ |  |  |  | Thrombozytopenie |  | Thrombozytopenie [M0777\|](http://85.214.20.219/memem7/Terms_List7.cfm?search=M0777\|) |  |  |  |  | [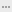](http://85.214.20.219/memem7/TermsPhemes_Edit.cfm?ppid=81273&concept=100C) |  |
| +++ |  |  |  | Clinical: Zytopenie |  | Zytopenie [M2451\|](http://85.214.20.219/memem7/Terms_List7.cfm?search=M2451\|) |  |  |  |  | [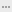](http://85.214.20.219/memem7/TermsPhemes_Edit.cfm?ppid=98447&concept=100C) |  |
| **Imaging** | | | | |  |  |  |  |  |  | [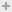](http://85.214.20.219/memem7/TermsPhemes_New.cfm?code7=M3101\|&cl=56&concept=100C) |  |
| **Pathologie** | | | | |  |  |  |  |  |  | [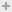](http://85.214.20.219/memem7/TermsPhemes_New.cfm?code7=M3101\|&cl=57&concept=100C) |  |
| +++ |  |  |  | Mikroskopie: Knochenmark: hyperzellulär |  | Knochenmark [O812\|](http://85.214.20.219/memem7/Terms_List7.cfm?search=O812\|) hohe Zelldichte [UB36\|](http://85.214.20.219/memem7/Terms_List7.cfm?search=UB36\|) |  |  |  |  | [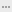](http://85.214.20.219/memem7/TermsPhemes_Edit.cfm?ppid=81280&concept=100C) |  |
| +++ |  |  |  | Mikroskopie: Knochenmark: Erythropoese: peritrabekulär |  | Knochenmark [O812\|](http://85.214.20.219/memem7/Terms_List7.cfm?search=O812\|) Erythropoese [ML86\|](http://85.214.20.219/memem7/Terms_List7.cfm?search=ML86\|) paratrabekulär [7959Z\|](http://85.214.20.219/memem7/Terms_List7.cfm?search=7959Z\|) |  |  |  |  | [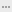](http://85.214.20.219/memem7/TermsPhemes_Edit.cfm?ppid=81281&concept=100C) |  |
| +++ |  |  |  | Mikroskopie: Knochenmark: Dyserythropoese |  | Knochenmark [O812\|](http://85.214.20.219/memem7/Terms_List7.cfm?search=O812\|) Dyserythropoese [10255T\|](http://85.214.20.219/memem7/Terms_List7.cfm?search=10255T\|) |  |  |  |  | [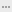](http://85.214.20.219/memem7/TermsPhemes_Edit.cfm?ppid=81282&concept=100C) |  |
| +++ |  |  |  | Mikroskopie: Knochenmark: Dysgranulopoese |  | Knochenmark [O812\|](http://85.214.20.219/memem7/Terms_List7.cfm?search=O812\|) Dysgranulopoese [10257T\|](http://85.214.20.219/memem7/Terms_List7.cfm?search=10257T\|) |  |  |  |  | [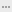](http://85.214.20.219/memem7/TermsPhemes_Edit.cfm?ppid=81283&concept=100C) |  |
| +++ |  |  |  | Mikroskopie: Knochenmark: Dysmegakaryopoese |  | Knochenmark [O812\|](http://85.214.20.219/memem7/Terms_List7.cfm?search=O812\|) Dysmegakaryopoese [10312T\|](http://85.214.20.219/memem7/Terms_List7.cfm?search=10312T\|) |  |  |  |  | [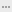](http://85.214.20.219/memem7/TermsPhemes_Edit.cfm?ppid=81284&concept=100C) |  |
| +++ |  |  |  | Mikroskopie: Knochenmark: Megakaryozyten: vermehrt |  | Knochenmark [O812\|](http://85.214.20.219/memem7/Terms_List7.cfm?search=O812\|) Knochenmarkriesenzelle [Z7213\|](http://85.214.20.219/memem7/Terms_List7.cfm?search=Z7213\|) Vermehrung [AE41\|](http://85.214.20.219/memem7/Terms_List7.cfm?search=AE41\|) |  |  |  |  | [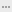](http://85.214.20.219/memem7/TermsPhemes_Edit.cfm?ppid=81293&concept=100C) |  |
| +++ |  |  |  | Mikroskopie: Knochenmark: Mikromegakaryozyten |  | Knochenmark [O812\|](http://85.214.20.219/memem7/Terms_List7.cfm?search=O812\|) Mikromegakaryozyt [10460T\|](http://85.214.20.219/memem7/Terms_List7.cfm?search=10460T\|) |  |  |  |  | [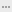](http://85.214.20.219/memem7/TermsPhemes_Edit.cfm?ppid=81294&concept=100C) |  |
| + |  |  |  | Mikroskopie: Knochenmark: normozellulär |  | Knochenmark [O812\|](http://85.214.20.219/memem7/Terms_List7.cfm?search=O812\|) mäßige Zelldichte [UB35\|](http://85.214.20.219/memem7/Terms_List7.cfm?search=UB35\|) |  |  |  |  | [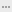](http://85.214.20.219/memem7/TermsPhemes_Edit.cfm?ppid=81295&concept=100C) |  |
| + |  |  |  | Mikroskopie: Knochenmark: hypozellulär |  | Knochenmark [O812\|](http://85.214.20.219/memem7/Terms_List7.cfm?search=O812\|) niedrige Zelldichte [UB34\|](http://85.214.20.219/memem7/Terms_List7.cfm?search=UB34\|) |  |  |  |  | [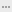](http://85.214.20.219/memem7/TermsPhemes_Edit.cfm?ppid=81296&concept=100C) |  |
| +++ |  |  |  | Mikroskopie: Knochenmark: Cluster: Blasten |  | Knochenmark [O812\|](http://85.214.20.219/memem7/Terms_List7.cfm?search=O812\|) Zellgruppe [10215T\|](http://85.214.20.219/memem7/Terms_List7.cfm?search=10215T\|) Blast [10217T\|](http://85.214.20.219/memem7/Terms_List7.cfm?search=10217T\|) |  |  |  |  | [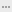](http://85.214.20.219/memem7/TermsPhemes_Edit.cfm?ppid=81297&concept=100C) |  |
| +++ |  |  |  | Mikroskopie: Knochenmark: abnormal localization of immature precursors |  | Knochenmark [O812\|](http://85.214.20.219/memem7/Terms_List7.cfm?search=O812\|) ALIP [10625T\|](http://85.214.20.219/memem7/Terms_List7.cfm?search=10625T\|) |  |  |  |  | [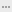](http://85.214.20.219/memem7/TermsPhemes_Edit.cfm?ppid=81298&concept=100C) |  |
| +++ |  |  |  | Mikroskopie: Knochenmark: ALIP |  | Knochenmark [O812\|](http://85.214.20.219/memem7/Terms_List7.cfm?search=O812\|) ALIP [10625T\|](http://85.214.20.219/memem7/Terms_List7.cfm?search=10625T\|) |  |  |  |  | [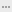](http://85.214.20.219/memem7/TermsPhemes_Edit.cfm?ppid=81299&concept=100C) |  |
| Lead | 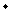 |  |  | Mikroskopie: Knochenmark: Myeloblasten 5 % |  | Knochenmark [O812\|](http://85.214.20.219/memem7/Terms_List7.cfm?search=O812\|) Myeloblast [10209T\|](http://85.214.20.219/memem7/Terms_List7.cfm?search=10209T\|) |  |  |  |  | [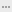](http://85.214.20.219/memem7/TermsPhemes_Edit.cfm?ppid=98018&concept=100C) |  |
| Lead | 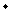 |  |  | Mikroskopie: Knochenmark: Myeloblasten 20 % |  | Knochenmark [O812\|](http://85.214.20.219/memem7/Terms_List7.cfm?search=O812\|) Myeloblast [10209T\|](http://85.214.20.219/memem7/Terms_List7.cfm?search=10209T\|) |  |  |  |  | [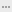](http://85.214.20.219/memem7/TermsPhemes_Edit.cfm?ppid=98019&concept=100C) |  |
| +++ |  |  |  | Mikroskopie: Knochenmark: zellreich |  | Knochenmark [O812\|](http://85.214.20.219/memem7/Terms_List7.cfm?search=O812\|) viele Zellen [UB12\|](http://85.214.20.219/memem7/Terms_List7.cfm?search=UB12\|) |  |  |  |  | [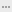](http://85.214.20.219/memem7/TermsPhemes_Edit.cfm?ppid=98448&concept=100C) |  |
| +++ |  |  |  | Mikroskopie: Knochenmark: ALIP: nachweisbar |  | Knochenmark [O812\|](http://85.214.20.219/memem7/Terms_List7.cfm?search=O812\|) ALIP [10625T\|](http://85.214.20.219/memem7/Terms_List7.cfm?search=10625T\|) nachweisbar [DU22\|](http://85.214.20.219/memem7/Terms_List7.cfm?search=DU22\|) |  |  |  |  | [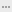](http://85.214.20.219/memem7/TermsPhemes_Edit.cfm?ppid=119255&concept=100C) |  |
| +++ |  |  |  | Zytologie: Blutausstrich: Myeloblast 2:19 %\| |  | Ausstrich peripheres Blut [10221T\|](http://85.214.20.219/memem7/Terms_List7.cfm?search=10221T\|) Myeloblast [10209T\|](http://85.214.20.219/memem7/Terms_List7.cfm?search=10209T\|) |  |  |  |  | [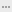](http://85.214.20.219/memem7/TermsPhemes_Edit.cfm?ppid=81274&concept=100C) |  |
| +++ |  |  |  | Zytologie: Blutausstrich: Erythrozyten: Anisozytose |  | Ausstrich peripheres Blut [10221T\|](http://85.214.20.219/memem7/Terms_List7.cfm?search=10221T\|) Erythrozyt [Z726\|](http://85.214.20.219/memem7/Terms_List7.cfm?search=Z726\|) Anisozytose [10627T\|](http://85.214.20.219/memem7/Terms_List7.cfm?search=10627T\|) |  |  |  |  | [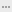](http://85.214.20.219/memem7/TermsPhemes_Edit.cfm?ppid=81275&concept=100C) |  |
| +++ |  |  |  | Zytologie: Blutausstrich: Erythrozyten: Poikilozytose |  | Ausstrich peripheres Blut [10221T\|](http://85.214.20.219/memem7/Terms_List7.cfm?search=10221T\|) Erythrozyt [Z726\|](http://85.214.20.219/memem7/Terms_List7.cfm?search=Z726\|) Poikilozytose [10629T\|](http://85.214.20.219/memem7/Terms_List7.cfm?search=10629T\|) |  |  |  |  | [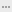](http://85.214.20.219/memem7/TermsPhemes_Edit.cfm?ppid=81276&concept=100C) |  |
| +++ |  |  |  | Zytologie: Blutausstrich: Thrombozyten: vergrößert |  | Ausstrich peripheres Blut [10221T\|](http://85.214.20.219/memem7/Terms_List7.cfm?search=10221T\|) Thrombozyt [Z780\|](http://85.214.20.219/memem7/Terms_List7.cfm?search=Z780\|) vergrößert [AG26\|](http://85.214.20.219/memem7/Terms_List7.cfm?search=AG26\|) |  |  |  |  | [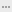](http://85.214.20.219/memem7/TermsPhemes_Edit.cfm?ppid=81277&concept=100C) |  |

Additional File 1: Table S2. RAEB part 2

| +++ |  |  |  | Zytologie: Blutausstrich: Thrombozyten: Granula: vermindert |  | Ausstrich peripheres Blut [10221T\|](http://85.214.20.219/memem7/Terms_List7.cfm?search=10221T\|) Thrombozyt [Z780\|](http://85.214.20.219/memem7/Terms_List7.cfm?search=Z780\|) Granula [10366T\|](http://85.214.20.219/memem7/Terms_List7.cfm?search=10366T\|) Verminderung [AE42\|](http://85.214.20.219/memem7/Terms_List7.cfm?search=AE42\|) |  |  |  |  | [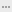](http://85.214.20.219/memem7/TermsPhemes_Edit.cfm?ppid=81278&concept=100C) |  |
| --- | --- | --- | --- | --- | --- | --- | --- | --- | --- | --- | --- | --- |
| +++ |  |  |  | Zytologie: Blutausstrich: neutrophile Granulozyten: Granula: abnormal |  | Ausstrich peripheres Blut [10221T\|](http://85.214.20.219/memem7/Terms_List7.cfm?search=10221T\|) segmentkerniger Granulozyt [Z742\|](http://85.214.20.219/memem7/Terms_List7.cfm?search=Z742\|) Granula [10366T\|](http://85.214.20.219/memem7/Terms_List7.cfm?search=10366T\|) Abnormalität [M3415\|](http://85.214.20.219/memem7/Terms_List7.cfm?search=M3415\|) |  |  |  |  | [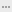](http://85.214.20.219/memem7/TermsPhemes_Edit.cfm?ppid=81279&concept=100C) |  |
| +++ |  |  |  | Zytologie: Knochenmark: Dyserythropoese: Erythroblasten: megaloblastoid |  | Knochenmark [O812\|](http://85.214.20.219/memem7/Terms_List7.cfm?search=O812\|) Dyserythropoese [10255T\|](http://85.214.20.219/memem7/Terms_List7.cfm?search=10255T\|) Erythroblast [Z7252\|](http://85.214.20.219/memem7/Terms_List7.cfm?search=Z7252\|) megaloblastoid [10372T\|](http://85.214.20.219/memem7/Terms_List7.cfm?search=10372T\|) |  |  |  |  | [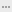](http://85.214.20.219/memem7/TermsPhemes_Edit.cfm?ppid=81285&concept=100C) |  |
| +++ |  |  |  | Zytologie: Knochenmark: Dyserythropoese: Erythroblasten: zweikernig |  | Knochenmark [O812\|](http://85.214.20.219/memem7/Terms_List7.cfm?search=O812\|) Dyserythropoese [10255T\|](http://85.214.20.219/memem7/Terms_List7.cfm?search=10255T\|) Erythroblast [Z7252\|](http://85.214.20.219/memem7/Terms_List7.cfm?search=Z7252\|) zweikernig [VF132\|](http://85.214.20.219/memem7/Terms_List7.cfm?search=VF132\|) |  |  |  |  | [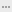](http://85.214.20.219/memem7/TermsPhemes_Edit.cfm?ppid=81286&concept=100C) |  |
| +++ |  |  |  | Zytologie: Knochenmark: Dyserythropoese: Erythroblasten: Brückenbildung: intranukleär |  | Knochenmark [O812\|](http://85.214.20.219/memem7/Terms_List7.cfm?search=O812\|) Dyserythropoese [10255T\|](http://85.214.20.219/memem7/Terms_List7.cfm?search=10255T\|) Erythroblast [Z7252\|](http://85.214.20.219/memem7/Terms_List7.cfm?search=Z7252\|) Brückenbildung [7100V\|](http://85.214.20.219/memem7/Terms_List7.cfm?search=7100V\|) intranukleär [3118Z\|](http://85.214.20.219/memem7/Terms_List7.cfm?search=3118Z\|) |  |  |  |  | [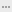](http://85.214.20.219/memem7/TermsPhemes_Edit.cfm?ppid=81287&concept=100C) | #? |
| +++ |  |  |  | Zytologie: Knochenmark: Dyserythropoese: Erythroblasten: Zellkerne: lobuliert |  | Knochenmark [O812\|](http://85.214.20.219/memem7/Terms_List7.cfm?search=O812\|) Dyserythropoese [10255T\|](http://85.214.20.219/memem7/Terms_List7.cfm?search=10255T\|) Erythroblast [Z7252\|](http://85.214.20.219/memem7/Terms_List7.cfm?search=Z7252\|) Zellkern [Y150\|](http://85.214.20.219/memem7/Terms_List7.cfm?search=Y150\|) gelappt [4761Z\|](http://85.214.20.219/memem7/Terms_List7.cfm?search=4761Z\|) |  |  |  |  | [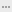](http://85.214.20.219/memem7/TermsPhemes_Edit.cfm?ppid=81288&concept=100C) | #? |
| +++ |  |  |  | Zytologie: Knochenmark: Dysgranulopoese: ähnlich: Pelger-Huet Anomalie |  | Knochenmark [O812\|](http://85.214.20.219/memem7/Terms_List7.cfm?search=O812\|) Dysgranulopoese [10257T\|](http://85.214.20.219/memem7/Terms_List7.cfm?search=10257T\|) Ähnlichkeit [7700V\|](http://85.214.20.219/memem7/Terms_List7.cfm?search=7700V\|) Pelger-Huët-Anomalie [M2047\|](http://85.214.20.219/memem7/Terms_List7.cfm?search=M2047\|) |  |  |  |  | [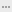](http://85.214.20.219/memem7/TermsPhemes_Edit.cfm?ppid=81289&concept=100C) |  |
| +++ |  |  |  | Zytologie: Knochenmark: Dysgranulopoese: Granula: vermindert |  | Knochenmark [O812\|](http://85.214.20.219/memem7/Terms_List7.cfm?search=O812\|) Dysgranulopoese [10257T\|](http://85.214.20.219/memem7/Terms_List7.cfm?search=10257T\|) Granula [10366T\|](http://85.214.20.219/memem7/Terms_List7.cfm?search=10366T\|) Verminderung [AE42\|](http://85.214.20.219/memem7/Terms_List7.cfm?search=AE42\|) |  |  |  |  | [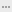](http://85.214.20.219/memem7/TermsPhemes_Edit.cfm?ppid=81290&concept=100C) |  |
| +++ |  |  |  | Zytologie: Knochenmark: Dysgranulopoese: Granula: ähnlich: Chediak-Higashi Syndrom |  | Knochenmark [O812\|](http://85.214.20.219/memem7/Terms_List7.cfm?search=O812\|) Dysgranulopoese [10257T\|](http://85.214.20.219/memem7/Terms_List7.cfm?search=10257T\|) Granula [10366T\|](http://85.214.20.219/memem7/Terms_List7.cfm?search=10366T\|) Ähnlichkeit [7700V\|](http://85.214.20.219/memem7/Terms_List7.cfm?search=7700V\|) Chediak-Higashi-Syndrom [M1843\|](http://85.214.20.219/memem7/Terms_List7.cfm?search=M1843\|) |  |  |  |  | [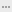](http://85.214.20.219/memem7/TermsPhemes_Edit.cfm?ppid=81291&concept=100C) | #? |
| +++ |  |  |  | Zytologie: Knochenmark: neutrophile Granulozyten: Zellgröße: vermindert |  | Knochenmark [O812\|](http://85.214.20.219/memem7/Terms_List7.cfm?search=O812\|) segmentkerniger Granulozyt [Z742\|](http://85.214.20.219/memem7/Terms_List7.cfm?search=Z742\|) Zellgröße [VA10\|](http://85.214.20.219/memem7/Terms_List7.cfm?search=VA10\|) Verminderung [AE42\|](http://85.214.20.219/memem7/Terms_List7.cfm?search=AE42\|) |  |  |  |  | [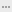](http://85.214.20.219/memem7/TermsPhemes_Edit.cfm?ppid=81292&concept=100C) |  |
| +++ |  |  |  | Immunhistochemie: CD61: Megakaryozyten: positiv |  | CD61 [11867L\|](http://85.214.20.219/memem7/Terms_List7.cfm?search=11867L\|) Knochenmarkriesenzelle [Z7213\|](http://85.214.20.219/memem7/Terms_List7.cfm?search=Z7213\|) nachweisbar [DU22\|](http://85.214.20.219/memem7/Terms_List7.cfm?search=DU22\|) |  |  |  |  | [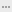](http://85.214.20.219/memem7/TermsPhemes_Edit.cfm?ppid=81313&concept=100C) |  |
| + |  |  |  | Immunhistochemie: CD42b: Megakaryozyten: positiv |  | CD42b [11829L\|](http://85.214.20.219/memem7/Terms_List7.cfm?search=11829L\|) Knochenmarkriesenzelle [Z7213\|](http://85.214.20.219/memem7/Terms_List7.cfm?search=Z7213\|) nachweisbar [DU22\|](http://85.214.20.219/memem7/Terms_List7.cfm?search=DU22\|) |  |  |  |  | [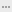](http://85.214.20.219/memem7/TermsPhemes_Edit.cfm?ppid=81314&concept=100C) |  |
| +++ |  |  |  | FACS: Zellen: positiv: CD34: nachweisbar |  | Körperzelle [Z0\|](http://85.214.20.219/memem7/Terms_List7.cfm?search=Z0\|) nachweisbar [DU22\|](http://85.214.20.219/memem7/Terms_List7.cfm?search=DU22\|) CD34 [11815L\|](http://85.214.20.219/memem7/Terms_List7.cfm?search=11815L\|) nachweisbar [DU22\|](http://85.214.20.219/memem7/Terms_List7.cfm?search=DU22\|) |  |  |  |  | [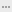](http://85.214.20.219/memem7/TermsPhemes_Edit.cfm?ppid=81305&concept=100C) |  |
| +++ |  |  |  | FACS: Zellen: positiv: CD117: nachweisbar |  | Körperzelle [Z0\|](http://85.214.20.219/memem7/Terms_List7.cfm?search=Z0\|) nachweisbar [DU22\|](http://85.214.20.219/memem7/Terms_List7.cfm?search=DU22\|) C-kit Protonokogen [11757L\|](http://85.214.20.219/memem7/Terms_List7.cfm?search=11757L\|) nachweisbar [DU22\|](http://85.214.20.219/memem7/Terms_List7.cfm?search=DU22\|) |  |  |  |  | [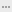](http://85.214.20.219/memem7/TermsPhemes_Edit.cfm?ppid=81306&concept=100C) |  |
| +++ |  |  |  | FACS: Immunphenotypus: CD34: positiv: AND: CD38: positiv: AND: CD13: positiv:AND: CD33: positiv |  | Immunphenotypus [10231T\|](http://85.214.20.219/memem7/Terms_List7.cfm?search=10231T\|) CD34 [11815L\|](http://85.214.20.219/memem7/Terms_List7.cfm?search=11815L\|) nachweisbar [DU22\|](http://85.214.20.219/memem7/Terms_List7.cfm?search=DU22\|) AND [.AND\|](http://85.214.20.219/memem7/Terms_List7.cfm?search=.AND\|) CD38 [11821L\|](http://85.214.20.219/memem7/Terms_List7.cfm?search=11821L\|) nachweisbar [DU22\|](http://85.214.20.219/memem7/Terms_List7.cfm?search=DU22\|) AND [.AND\|](http://85.214.20.219/memem7/Terms_List7.cfm?search=.AND\|) Aminopeptidase IV [11770L\|](http://85.214.20.219/memem7/Terms_List7.cfm?search=11770L\|) nachweisbar [DU22\|](http://85.214.20.219/memem7/Terms_List7.cfm?search=DU22\|) AND [.AND\|](http://85.214.20.219/memem7/Terms_List7.cfm?search=.AND\|) CD33 [11813L\|](http://85.214.20.219/memem7/Terms_List7.cfm?search=11813L\|) nachweisbar [DU22\|](http://85.214.20.219/memem7/Terms_List7.cfm?search=DU22\|) [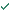](http://85.214.20.219/memem7/TermsPhemes_FixUpdate.cfm?ppid=81307&code7=M3101\|) |  |  |  |  | [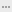](http://85.214.20.219/memem7/TermsPhemes_Edit.cfm?ppid=81307&concept=100C) | #? |
| +++ |  |  |  | FACS: Myeloblasten: CD15: positiv |  | Myeloblast [10209T\|](http://85.214.20.219/memem7/Terms_List7.cfm?search=10209T\|) CD15 [11775L\|](http://85.214.20.219/memem7/Terms_List7.cfm?search=11775L\|) nachweisbar [DU22\|](http://85.214.20.219/memem7/Terms_List7.cfm?search=DU22\|) |  |  | 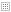 |  | [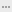](http://85.214.20.219/memem7/TermsPhemes_Edit.cfm?ppid=81308&concept=100C) |  |

Additional File 1: Table S3. RAEB part 3

| +++ |  |  |  | FACS: Myeloblasten: CD11b: positiv |  | Myeloblast [10209T\|](http://85.214.20.219/memem7/Terms_List7.cfm?search=10209T\|) CD11b [11760L\|](http://85.214.20.219/memem7/Terms_List7.cfm?search=11760L\|) nachweisbar [DU22\|](http://85.214.20.219/memem7/Terms_List7.cfm?search=DU22\|) |  |  | 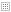 |  | [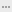](http://85.214.20.219/memem7/TermsPhemes_Edit.cfm?ppid=81309&concept=100C) |  |
| --- | --- | --- | --- | --- | --- | --- | --- | --- | --- | --- | --- | --- |
| +++ |  |  |  | FACS: Myeloblasten: CD65: positiv |  | Myeloblast [10209T\|](http://85.214.20.219/memem7/Terms_List7.cfm?search=10209T\|) CD65 [52003L\|](http://85.214.20.219/memem7/Terms_List7.cfm?search=52003L\|) nachweisbar [DU22\|](http://85.214.20.219/memem7/Terms_List7.cfm?search=DU22\|) |  |  | 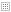 |  | [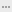](http://85.214.20.219/memem7/TermsPhemes_Edit.cfm?ppid=81310&concept=100C) |  |
| +++ | 10% |  |  | FACS: Myeloblasten: CD7: positiv |  | Myeloblast [10209T\|](http://85.214.20.219/memem7/Terms_List7.cfm?search=10209T\|) CD7 [11882L\|](http://85.214.20.219/memem7/Terms_List7.cfm?search=11882L\|) nachweisbar [DU22\|](http://85.214.20.219/memem7/Terms_List7.cfm?search=DU22\|) |  |  | 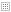 |  | [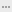](http://85.214.20.219/memem7/TermsPhemes_Edit.cfm?ppid=81311&concept=100C) |  |
| +++ | 20% |  |  | FACS: Myeloblasten: CD56: positiv |  | Myeloblast [10209T\|](http://85.214.20.219/memem7/Terms_List7.cfm?search=10209T\|) CD56 [11860L\|](http://85.214.20.219/memem7/Terms_List7.cfm?search=11860L\|) nachweisbar [DU22\|](http://85.214.20.219/memem7/Terms_List7.cfm?search=DU22\|) |  |  | 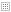 |  | [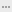](http://85.214.20.219/memem7/TermsPhemes_Edit.cfm?ppid=81312&concept=100C) |  |
| **Genetik** | | | | |  |  |  |  |  |  | [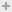](http://85.214.20.219/memem7/TermsPhemes_New.cfm?code7=M3101\|&cl=58&concept=100C) |  |
|  | 30-50% |  |  | Aberration: genetisch |  | Fehlentwicklung [7749V\|](http://85.214.20.219/memem7/Terms_List7.cfm?search=7749V\|) Erbanlage [1850Z\|](http://85.214.20.219/memem7/Terms_List7.cfm?search=1850Z\|) |  |  |  |  | [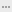](http://85.214.20.219/memem7/TermsPhemes_Edit.cfm?ppid=81315&concept=100C) |  |
|  |  |  |  | Chromosom +8 |  | Chromosom [51849L\|](http://85.214.20.219/memem7/Terms_List7.cfm?search=51849L\|) |  |  |  |  |  |  |
|  |  |  |  | Chromosom -5 |  | Chromosom [51849L\|](http://85.214.20.219/memem7/Terms_List7.cfm?search=51849L\|) |  |  |  |  |  |  |
|  |  |  |  | genetische Aberration del5q |  | genetische Aberration [4762Z\|](http://85.214.20.219/memem7/Terms_List7.cfm?search=4762Z\|) |  |  |  |  |  |  |
|  |  |  |  | Deletion: Chromosom -7 |  | Deletion [52074L\|](http://85.214.20.219/memem7/Terms_List7.cfm?search=52074L\|) Chromosom [51849L\|](http://85.214.20.219/memem7/Terms_List7.cfm?search=51849L\|) |  |  |  |  |  |  |
| **Psychologie** | | | | |  |  |  |  |  |  |  |  |
|  |  |  |  |  |  |  |  |  |  |  |  |  |
| **Characteristics** | | | | |  | [Code](http://85.214.20.219/memem7/TermsPhemes_TransUpdate.cfm?code7=M3101\|&cl=6&concept=100C) |  |  |  |  |  |  |
| **Historie** | | | | | | | | | | |  |  |
| **Epidemologie** | | | | |  |  |  |  |  |  |  |  |
|  | 40% |  |  | Myelodysplasie |  | Myelodysplasie [M1330\|](http://85.214.20.219/memem7/Terms_List7.cfm?search=M1330\|) |  |  |  |  |  |  |
| +++ |  |  |  | Age: Alter >50 Jahre |  | Alter [BB\|](http://85.214.20.219/memem7/Terms_List7.cfm?search=BB\|) |  |  |  |  |  |  |
| **Ätiologie** | | | | |  |  |  |  |  |  |  |  |
| **Pathophysiologie** | | | | |  |  |  |  |  |  |  |  |
| **Verlauf** | | | | |  |  |  |  |  |  |  |  |
| Lead |  |  |  | Verlauf: chronisch |  | chronisch [DE20\|](http://85.214.20.219/memem7/Terms_List7.cfm?search=DE20\|) |  |  |  |  |  |  |
| +++ |  |  |  | Stadium: IPSS Score |  | [**IPSS Score ??\|**](http://85.214.20.219/memem7/Terms_List.cfm?search=IPSS%20Score&ppid=81323) |  |  |  |  |  |  |
| +++ |  |  |  | Stadium: mittleres Überleben: if: RAEB-2 9 Monate |  | mittleres Überleben [7832V\|](http://85.214.20.219/memem7/Terms_List7.cfm?search=7832V\|) WHEN [8576V\|](http://85.214.20.219/memem7/Terms_List7.cfm?search=8576V\|) RAEB-2 [M3112\|](http://85.214.20.219/memem7/Terms_List7.cfm?search=M3112\|) |  |  |  |  |  |  |
| +++ |  |  |  | Prognose: mittleres Überleben: if: RAEB-1 16 Monate |  | mittleres Überleben [7832V\|](http://85.214.20.219/memem7/Terms_List7.cfm?search=7832V\|) WHEN [8576V\|](http://85.214.20.219/memem7/Terms_List7.cfm?search=8576V\|) RAEB-1 [M3110\|](http://85.214.20.219/memem7/Terms_List7.cfm?search=M3110\|) |  |  |  |  |  |  |
| ++ | 25% |  |  | Komplikation: AML |  | Akute myeloische Leukämie [M1179\|](http://85.214.20.219/memem7/Terms_List7.cfm?search=M1179\|) |  |  |  |  |  |  |
| ++ | 33% |  |  | Komplikation: AML |  | Akute myeloische Leukämie [M1179\|](http://85.214.20.219/memem7/Terms_List7.cfm?search=M1179\|) |  |  |  |  |  |  |
| **Komorbidität** | | | | |  |  |  |  |  |  |  |  |
| **Differentialdiagnose** | | | | |  |  |  |  |  |  |  |  |
| +++ |  |  |  | RCUD |  | refraktäre Anämie mit unilineärer Dyslasie [M1457\|](http://85.214.20.219/memem7/Terms_List7.cfm?search=M1457\|) |  |  |  |  |  |  |
| +++ |  |  |  | RCMD |  | refrakäre Zytopenie mit multilineärer Dysplasie [M3100\|](http://85.214.20.219/memem7/Terms_List7.cfm?search=M3100\|) |  |  |  |  |  |  |
| +++ |  |  |  | AML |  | Akute myeloische Leukämie [M1179\|](http://85.214.20.219/memem7/Terms_List7.cfm?search=M1179\|) |  |  |  |  |  |  |
| ++ |  |  |  | RARS |  | Refraktäre Anämie mit Ringsideroblasten [75564R\|](http://85.214.20.219/memem7/Terms_List7.cfm?search=75564R\|) |  |  |  |  |  |  |

Additional File 1: Table S4. RAEB part 4. Note that the underscored pathophem (IPSS Score) is not yet included in the system.

| **Term** | *Lang* | *Code7* |  | *Class* | *Num* | *Prag* | *Order* | *Gen* | *Kas* | *Tem* | *Fm* | *#P* |  | Formularbeginn  Formularende |
| --- | --- | --- | --- | --- | --- | --- | --- | --- | --- | --- | --- | --- | --- | --- |
| inflammatorisches Mammakarzinom | dt | [M5729\|](http://85.214.20.219/memem7/Terms_List7.cfm?search=M5729\|) |  | Subst |  |  | 1 |  |  |  |  | [1](http://85.214.20.219/memem7/TermsPhemes_List.cfm?link7=M5729\|&concept=100C) |  |  |
| inflammatory breast cancer | eng | [M5729\|](http://85.214.20.219/memem7/Terms_List7.cfm?search=M5729\|) |  | Subst |  |  |  |  |  |  |  |  |  |  |
| [ICD C50.-](http://85.214.20.219/memem7/ICDX_List.cfm?icx=C50.-) |  | [M5729\|](http://85.214.20.219/memem7/Terms_List7.cfm?search=M5729\|) |  | Code |  | | | | | | | |  |  |
| [ICD-O 8530/3](http://85.214.20.219/memem7/ICO_List.cfm?search=8530/3) |  | [M5729\|](http://85.214.20.219/memem7/Terms_List7.cfm?search=M5729\|) |  | Code |  | | | | | | | |  |  |

|  | | | | |  |  |
| --- | --- | --- | --- | --- | --- | --- |
|  |  |  |  |  |  |  |
| **Definition** *Texte / Web / Media* | | *Type* | | |  |  |
|  | | | | |  |  |

|  | | | | | | | | | | |  |  |
| --- | --- | --- | --- | --- | --- | --- | --- | --- | --- | --- | --- | --- |
| **Net** |  | *Link1* | *Link2* | *Link3* | *Val* | | | | *Type* | |  |  |
| **Meta** | | | | | | | | | | |  |  |
| inflammatorisches Mammakarzinom [M5729\|](http://85.214.20.219/memem7/Terms_List7.cfm?search=M5729\|) |  | Krankheit[DA01\|](http://85.214.20.219/memem7/Terms_List7.cfm?search=DA01\|) |  |  |  |  |  |  | meta |  |  |  |
| inflammatorisches Mammakarzinom [M5729\|](http://85.214.20.219/memem7/Terms_List7.cfm?search=M5729\|) |  | Pathosom[M7\|](http://85.214.20.219/memem7/Terms_List7.cfm?search=M7\|) |  | PF[/2\|](http://85.214.20.219/memem7/Terms_List7.cfm?search=/2\|) |  |  |  |  | meta |  |  |  |

|  | | | | | | | | | | |  |  |
| --- | --- | --- | --- | --- | --- | --- | --- | --- | --- | --- | --- | --- |
|  |  |  |  |  |  |  |  |  |  |  |  |  |
| **Description** | | | | |  | [Code](http://85.214.20.219/memem7/TermsPhemes_TransUpdate.cfm?code7=M5729\|&cl=1&concept=100C) |  |  |  |  |  |  |
| **Definition** | | | | | | | | | | |  |  |
| **System/Lokalisation** | | | | |  |  |  |  |  |  |  |  |
| Lead |  |  |  | Mamma |  | Mamma [O570\|](http://85.214.20.219/memem7/Terms_List7.cfm?search=O570\|) |  |  |  |  |  |  |
| Lead |  |  |  | Lymphgefäß |  | Lymphgefäß [O880\|](http://85.214.20.219/memem7/Terms_List7.cfm?search=O880\|) |  |  |  |  |  |  |
| **Struktur** | | | | |  |  |  |  |  |  |  |  |
| Lead |  |  |  | ElementOf: invasives Mammakarzinom |  | invasives Mammakarzinom [M5370\|](http://85.214.20.219/memem7/Terms_List7.cfm?search=M5370\|) |  |  |  |  |  |  |
|  |  |  |  |  |  |  |  |  |  |  |  |  |
| **Symptoms** | | | | |  | [Code](http://85.214.20.219/memem7/TermsPhemes_TransUpdate.cfm?code7=M5729\|&cl=5&concept=100C) |  |  |  |  |  |  |
| **Anamnese** | | | | | | | | | | |  |  |
| +++ |  |  |  | Vergrößerung: Mamma |  | vergrößert [AG26\|](http://85.214.20.219/memem7/Terms_List7.cfm?search=AG26\|) Mamma [O570\|](http://85.214.20.219/memem7/Terms_List7.cfm?search=O570\|) |  |  |  |  |  |  |
| +++ |  |  |  | Rötung: Mamma |  | Rötung [SA31\|](http://85.214.20.219/memem7/Terms_List7.cfm?search=SA31\|) Mamma [O570\|](http://85.214.20.219/memem7/Terms_List7.cfm?search=O570\|) |  |  |  |  |  |  |
| +++ |  |  |  | Orangenhaut |  | Orangenhaut [7984V\|](http://85.214.20.219/memem7/Terms_List7.cfm?search=7984V\|) |  |  |  |  |  |  |
| +++ |  |  |  | Akut: Mamma: Tastbefund: fest |  | Mamma [O570\|](http://85.214.20.219/memem7/Terms_List7.cfm?search=O570\|) Tastbefund [8022V\|](http://85.214.20.219/memem7/Terms_List7.cfm?search=8022V\|) Festigkeit [AM50\|](http://85.214.20.219/memem7/Terms_List7.cfm?search=AM50\|) |  |  |  |  |  |  |
| **Vital** | | | | |  |  |  |  |  |  |  |  |
| **Physikal** | | | | |  |  |  |  |  |  |  |  |
| **Labor** | | | | |  |  |  |  |  |  |  |  |
| **Imaging** | | | | |  |  |  |  |  |  |  |  |
| **Pathologie** | | | | |  |  |  |  |  |  |  |  |
| Lead |  |  |  | pT-Klassifikation pT4d |  | TNM-Klassifikation T [5044Z\|](http://85.214.20.219/memem7/Terms_List7.cfm?search=5044Z\|) |  |  |  |  |  |  |
| Lead |  |  |  | Mikroskopie: Lymphangiosis carcinomatosa |  | Lymphangiosis carcinomatosa [6902V\|](http://85.214.20.219/memem7/Terms_List7.cfm?search=6902V\|) |  |  |  |  |  |  |
| Lead |  |  |  | Mikroskopie: Befall: Lymphgefäße |  | Befall [DC21\|](http://85.214.20.219/memem7/Terms_List7.cfm?search=DC21\|) Lymphgefäß [O880\|](http://85.214.20.219/memem7/Terms_List7.cfm?search=O880\|) |  |  |  |  |  |  |
| +++ |  |  |  | Mikroskopie: invasives duktales Mammakarzinom |  | Duktales Adenokarzinom [M0633\|](http://85.214.20.219/memem7/Terms_List7.cfm?search=M0633\|) |  |  |  |  |  |  |
| +++ |  |  |  | Mikroskopie: Angiosis carcinomatosa |  | Angiosis carcinomatosa [4226Z\|](http://85.214.20.219/memem7/Terms_List7.cfm?search=4226Z\|) |  |  |  |  |  |  |
|  | 50% |  |  | Zytologie: Progesteronrezeptor: positiv |  | progesterone receptor [17046L\|](http://85.214.20.219/memem7/Terms_List7.cfm?search=17046L\|) nachweisbar [DU22\|](http://85.214.20.219/memem7/Terms_List7.cfm?search=DU22\|) |  |  |  |  |  |  |
| +++ |  |  |  | Zytologie: E-Cadherin: positiv |  | E-Cadherin [52164L\|](http://85.214.20.219/memem7/Terms_List7.cfm?search=52164L\|) nachweisbar [DU22\|](http://85.214.20.219/memem7/Terms_List7.cfm?search=DU22\|) |  |  |  |  |  |  |
|  | 50% |  |  | Immunhistochemie: Östrogenrezeptor: positiv |  | estrogen receptor [13375L\|](http://85.214.20.219/memem7/Terms_List7.cfm?search=13375L\|) nachweisbar [DU22\|](http://85.214.20.219/memem7/Terms_List7.cfm?search=DU22\|) |  |  |  |  |  |  |
| ++ | 40% |  |  | Immunhistochemie: c-erbB2: positiv |  | HER2 [14185L\|](http://85.214.20.219/memem7/Terms_List7.cfm?search=14185L\|) nachweisbar [DU22\|](http://85.214.20.219/memem7/Terms_List7.cfm?search=DU22\|) |  |  |  |  |  |  |
| ++ |  |  |  | Immunhistochemie: EGFR: positiv |  | epidermal growth factor receptor [3236H\|](http://85.214.20.219/memem7/Terms_List7.cfm?search=3236H\|) nachweisbar [DU22\|](http://85.214.20.219/memem7/Terms_List7.cfm?search=DU22\|) |  |  |  |  |  |  |
| +++ |  |  |  | Immunhistochemie: MUC1: positiv |  | epitheliales membranes Antigen [51740L\|](http://85.214.20.219/memem7/Terms_List7.cfm?search=51740L\|) nachweisbar [DU22\|](http://85.214.20.219/memem7/Terms_List7.cfm?search=DU22\|) |  |  |  |  |  |  |

Additional File 1: Table S5. inflammatory breast cancer part 1

| +++ |  |  |  | Immunhistochemie: p53: positiv |  | p53 [20096L\|](http://85.214.20.219/memem7/Terms_List7.cfm?search=20096L\|) nachweisbar [DU22\|](http://85.214.20.219/memem7/Terms_List7.cfm?search=DU22\|) |  |  |  |  |  |  |
| --- | --- | --- | --- | --- | --- | --- | --- | --- | --- | --- | --- | --- |
| +++ |  |  |  | Immunhistochemie: E-Cadherin: positiv |  | E-Cadherin [52164L\|](http://85.214.20.219/memem7/Terms_List7.cfm?search=52164L\|) nachweisbar [DU22\|](http://85.214.20.219/memem7/Terms_List7.cfm?search=DU22\|) |  |  |  |  |  |  |
| **Genetik** | | | | |  |  |  |  |  |  |  |  |
| +++ | 90% |  |  | Überexpression: Rho-GTPase |  | Überexpression [5500Z\|](http://85.214.20.219/memem7/Terms_List7.cfm?search=5500Z\|) Rho-GTPase [7830Z\|](http://85.214.20.219/memem7/Terms_List7.cfm?search=7830Z\|) |  |  |  |  |  |  |
| +++ |  |  |  | Mutation: p53 |  | Mutation [7531V\|](http://85.214.20.219/memem7/Terms_List7.cfm?search=7531V\|) p53 [20096L\|](http://85.214.20.219/memem7/Terms_List7.cfm?search=20096L\|) |  |  |  |  |  |  |
| +++ |  |  |  | Amplifikation: Gen: c-erb-B2 |  | amplification [9097Z\|](http://85.214.20.219/memem7/Terms_List7.cfm?search=9097Z\|) Erbanlage [1850Z\|](http://85.214.20.219/memem7/Terms_List7.cfm?search=1850Z\|) HER2 [14185L\|](http://85.214.20.219/memem7/Terms_List7.cfm?search=14185L\|) |  |  |  |  |  |  |
| +++ |  |  |  | Alteration: Gen: anaplastic lymphoma kinase |  | Läsion [EK11\|](http://85.214.20.219/memem7/Terms_List7.cfm?search=EK11\|) Erbanlage [1850Z\|](http://85.214.20.219/memem7/Terms_List7.cfm?search=1850Z\|) ALK [52143L\|](http://85.214.20.219/memem7/Terms_List7.cfm?search=52143L\|) |  |  |  |  |  |  |
| **Psychologie** | | | | |  |  |  |  |  |  |  |  |
|  |  |  |  |  |  |  |  |  |  |  |  |  |
| **Characteristics** | | | | |  | [Code](http://85.214.20.219/memem7/TermsPhemes_TransUpdate.cfm?code7=M5729\|&cl=6&concept=100C) |  |  |  |  |  |  |
| **Historie** | | | | | | | | | | |  |  |
| **Epidemologie** | | | | |  |  |  |  |  |  |  |  |
| +++ | 99% |  |  | Sex: weiblich |  | Frau [BA12\|](http://85.214.20.219/memem7/Terms_List7.cfm?search=BA12\|) |  |  |  |  |  |  |
| +++ |  |  |  | Race: Afroamerikaner |  | Afroamerikaner [5499Z\|](http://85.214.20.219/memem7/Terms_List7.cfm?search=5499Z\|) |  |  |  |  |  |  |
|  |  |  |  | Inzidenz: Inzidenz 1/10^5 /anno |  | Inzidenz [3706Z\|](http://85.214.20.219/memem7/Terms_List7.cfm?search=3706Z\|) |  |  |  |  |  |  |
|  | 1% |  |  | Prävalenz: invasives Mammakarzinom |  | invasives Mammakarzinom [M5370\|](http://85.214.20.219/memem7/Terms_List7.cfm?search=M5370\|) |  |  |  |  |  |  |
| **Ätiologie** | | | | |  |  |  |  |  |  |  |  |
| ++ |  |  |  | Verlust: WISP3: CCN6 |  | Verlust [DF59\|](http://85.214.20.219/memem7/Terms_List7.cfm?search=DF59\|) WISP3 [52365L\|](http://85.214.20.219/memem7/Terms_List7.cfm?search=52365L\|) WISP3 [52365L\|](http://85.214.20.219/memem7/Terms_List7.cfm?search=52365L\|) |  |  |  |  |  |  |
| ++ |  |  |  | Überexpression: EIF4G1 |  | Überexpression [5500Z\|](http://85.214.20.219/memem7/Terms_List7.cfm?search=5500Z\|) eukaryotic translation initiation factor 4 gamma, 1 [3296H\|](http://85.214.20.219/memem7/Terms_List7.cfm?search=3296H\|) |  |  |  |  |  |  |
| **Pathophysiologie** | | | | |  |  |  |  |  |  |  |  |
| **Verlauf** | | | | |  |  |  |  |  |  |  |  |
| +++ |  |  |  | Stadium: TNM-Klassifikation T4d |  | TNM Klassifikation [2115Z\|](http://85.214.20.219/memem7/Terms_List7.cfm?search=2115Z\|) |  |  |  |  |  |  |
| +++ | 75% |  |  | Prognose: Letalität |  | Letalität [8537V\|](http://85.214.20.219/memem7/Terms_List7.cfm?search=8537V\|) |  |  |  |  |  |  |
| +++ | 50% |  |  | Prognose: Letalität: neoadjuvante Chemotherapie |  | Letalität [8537V\|](http://85.214.20.219/memem7/Terms_List7.cfm?search=8537V\|) neoadjuvante Chemotherapie [11128Z\|](http://85.214.20.219/memem7/Terms_List7.cfm?search=11128Z\|) |  |  |  |  |  |  |
| +++ |  |  |  | Risikofaktor: BMI: erhöht |  | Body Mass Index [2527Z\|](http://85.214.20.219/memem7/Terms_List7.cfm?search=2527Z\|) erhöhte Werte [DU12\|](http://85.214.20.219/memem7/Terms_List7.cfm?search=DU12\|) |  |  |  |  |  |  |
| **Komorbidität** | | | | |  |  |  |  |  |  |  |  |
| **Differentialdiagnose** | | | | |  |  |  |  |  |  |  |  |
| +++ |  |  |  | akute Mastitis |  | Akute Mastitis [M3260\|](http://85.214.20.219/memem7/Terms_List7.cfm?search=M3260\|) |  |  |  |  |  |  |
| +++ |  |  |  | Abszeß: Mamma |  | Abszess [EN21\|](http://85.214.20.219/memem7/Terms_List7.cfm?search=EN21\|) Mamma [O570\|](http://85.214.20.219/memem7/Terms_List7.cfm?search=O570\|) |  |  |  |  |  |  |
| **Untersuchung** | | | | |  |  |  |  |  |  |  |  |
|  |  |  |  |  |  |  |  |  |  |  |  |  |
| **Therapy** | | | | |  | [Code](http://85.214.20.219/memem7/TermsPhemes_TransUpdate.cfm?code7=M5729\|&cl=8&concept=100C) |  |  |  |  |  |  |
| **Therapieprinzipien** | | | | | | | | | | |  |  |
| **Medikamente** | | | | |  |  |  |  |  |  |  |  |

Additional File 1: Table S6. inflammatory breast cancer part 2

| membranoproliferative GN | | | | | | | | | | | | |  |  |
| --- | --- | --- | --- | --- | --- | --- | --- | --- | --- | --- | --- | --- | --- | --- |
| **Term** | *Lang* | *Code7* |  | *Class* | *Num* | *Prag* | *Order* | *Gen* | *Kas* | *Tem* | *Fm* | *#P* |  |  |
| membranoproliferative GN | dt | [M5032\|](http://85.214.20.219/memem7/Terms_List7.cfm?search=M5032\|) |  | Subst |  |  | 1 |  |  |  |  | [16](http://85.214.20.219/memem7/TermsPhemes_List.cfm?link7=M5032\|&concept=100C) |  |  |
| Glomerulonephritis, mesangiokapilläre | dt | [M5032\|](http://85.214.20.219/memem7/Terms_List7.cfm?search=M5032\|) |  | Subst |  |  |  |  |  |  |  |  |  |  |
| Membranoproliferative Glomerulonephritis | dt | [M5032\|](http://85.214.20.219/memem7/Terms_List7.cfm?search=M5032\|) |  | Subst |  |  |  |  |  |  | c |  |  |  |
| membrano-proliferative Glomerulonephritis | dt | [M5032\|](http://85.214.20.219/memem7/Terms_List7.cfm?search=M5032\|) |  | Subst |  |  |  |  |  |  |  |  |  |  |
| mesangiokapilläre Glomerulonephritis | dt | [M5032\|](http://85.214.20.219/memem7/Terms_List7.cfm?search=M5032\|) |  | Subst |  |  |  |  |  |  |  |  |  |  |
| mesangio-proliferative Glomerulonephritis | dt | [M5032\|](http://85.214.20.219/memem7/Terms_List7.cfm?search=M5032\|) |  | Subst |  |  |  |  |  |  |  |  |  |  |
| N04.5 | dt | [M5032\|](http://85.214.20.219/memem7/Terms_List7.cfm?search=M5032\|) |  | Subst |  |  |  |  |  |  |  |  |  |  |
| MPGN | dt | [M5032\|](http://85.214.20.219/memem7/Terms_List7.cfm?search=M5032\|) |  | Akronym |  | | | | | | | |  |  |
| [ICD N02.5](http://85.214.20.219/memem7/ICDX_List.cfm?icx=N02.5) |  | [M5032\|](http://85.214.20.219/memem7/Terms_List7.cfm?search=M5032\|) |  | Code |  | | | | | | | |  |  |
| [ICD N05.5](http://85.214.20.219/memem7/ICDX_List.cfm?icx=N05.5) |  | [M5032\|](http://85.214.20.219/memem7/Terms_List7.cfm?search=M5032\|) |  | Code |  | | | | | | | |  |  |
| [OMIM 305800](http://www.omim.org/entry/305800) |  | [M5032\|](http://85.214.20.219/memem7/Terms_List7.cfm?search=M5032\|) |  | Code |  | | | | | | | |  |  |
| [OMIM 609814](http://www.omim.org/entry/609814) |  | [M5032\|](http://85.214.20.219/memem7/Terms_List7.cfm?search=M5032\|) |  | Code |  | | | | | | | |  |  |
| [OMIM 614809](http://www.omim.org/entry/614809) |  | [M5032\|](http://85.214.20.219/memem7/Terms_List7.cfm?search=M5032\|) |  | Code |  | | | | | | | |  |  |
| [OMIM 615008](http://www.omim.org/entry/615008) |  | [M5032\|](http://85.214.20.219/memem7/Terms_List7.cfm?search=M5032\|) |  | Code |  | | | | | | | |  |  |
| [ORPHA 54370](http://www.orpha.net/consor/cgi-bin/OC_Exp.php?lng=DE&Expert=54370) |  | [M5032\|](http://85.214.20.219/memem7/Terms_List7.cfm?search=M5032\|) |  | Code |  | | | | | | | |  |  |

|  | | | | |  |  |
| --- | --- | --- | --- | --- | --- | --- |
|  |  |  |  |  |  |  |
| **Definition** *Texte / Web / Media* | | *Type* | | |  |  |
|  | Google Images: membrano-proliferative GN | web |  |  |  |  |
|  | | | | |  |  |

|  | | | | | | | | | | |  |  |
| --- | --- | --- | --- | --- | --- | --- | --- | --- | --- | --- | --- | --- |
| **Net** |  | *Link1* | *Link2* | *Link3* | *Val* | | | | *Type* | |  |  |
| **Meta** | | | | | | | | | | |  |  |
| membranoproliferative GN [M5032\|](http://85.214.20.219/memem7/Terms_List7.cfm?search=M5032\|) |  | Krankheit[DA01\|](http://85.214.20.219/memem7/Terms_List7.cfm?search=DA01\|) |  |  |  |  |  |  | meta |  |  |  |
| membranoproliferative GN [M5032\|](http://85.214.20.219/memem7/Terms_List7.cfm?search=M5032\|) |  | Pathosom[M7\|](http://85.214.20.219/memem7/Terms_List7.cfm?search=M7\|) |  | PF[/2\|](http://85.214.20.219/memem7/Terms_List7.cfm?search=/2\|) |  |  |  |  | meta |  |  |  |

|  | | | | | | | | | | |  |  |
| --- | --- | --- | --- | --- | --- | --- | --- | --- | --- | --- | --- | --- |
|  |  |  |  |  |  |  |  |  |  |  |  |  |
| **Description** | | | | |  | [Code](http://85.214.20.219/memem7/TermsPhemes_TransUpdate.cfm?code7=M5032\|&cl=1&concept=100C) |  |  |  |  |  |  |
| **Definition** | | | | | | | | | | |  |  |
| **System/Lokalisation** | | | | |  |  |  |  |  |  |  |  |
| **Struktur** | | | | |  |  |  |  |  |  |  |  |
| Lead |  |  |  | ElementOf: Glomerulonephritis |  | Glomerulonephritis [M2059\|](http://85.214.20.219/memem7/Terms_List7.cfm?search=M2059\|) |  |  |  |  |  |  |
| +++ |  |  |  | ElementOf: Immunkomplexkrankheit |  | Immunkomplexkrankheit [6401Z\|](http://85.214.20.219/memem7/Terms_List7.cfm?search=6401Z\|) |  |  |  |  |  |  |
| +++ |  |  |  | ElementOf: Erkrankung: Komplementsystem |  | Krankheit [DA01\|](http://85.214.20.219/memem7/Terms_List7.cfm?search=DA01\|) complement [51898L\|](http://85.214.20.219/memem7/Terms_List7.cfm?search=51898L\|) |  |  |  |  |  |  |
| +++ |  |  |  | HasElement: dense deposit disease |  | MPGN Typ II [M5292\|](http://85.214.20.219/memem7/Terms_List7.cfm?search=M5292\|) |  |  |  |  |  |  |
| +++ |  |  |  | HasVariante: primäre Form |  | primäre Form [2620Z\|](http://85.214.20.219/memem7/Terms_List7.cfm?search=2620Z\|) |  |  |  |  |  |  |
| +++ |  |  |  | HasVariante: sekundäre Form |  | sekundäre Form [7570V\|](http://85.214.20.219/memem7/Terms_List7.cfm?search=7570V\|) |  |  |  |  |  |  |
| +++ |  |  |  | HasVariante: MPGN Typ I |  | MPGN Typ I [M5880\|](http://85.214.20.219/memem7/Terms_List7.cfm?search=M5880\|) |  |  |  |  |  |  |
| +++ |  |  |  | HasVariante: MPGN Typ II |  | MPGN Typ II [M5292\|](http://85.214.20.219/memem7/Terms_List7.cfm?search=M5292\|) |  |  |  |  |  |  |
| +++ |  |  |  | HasVariante: MPGN Typ III |  | MPGN Typ III [M5881\|](http://85.214.20.219/memem7/Terms_List7.cfm?search=M5881\|) |  |  |  |  |  |  |
| +++ |  |  |  | HasVariante: C3-Glomerulonephritis |  | C3-Glomerulonephritis [M5293\|](http://85.214.20.219/memem7/Terms_List7.cfm?search=M5293\|) |  |  |  |  |  |  |
|  |  |  |  |  |  |  |  |  |  |  |  |  |

Additional File 1: Table S7. membrano-proliferative glomerulonephritis part 1

| \| **Anamnese** \|  \|  \| \| --- \| --- \| --- \| \| **Vital** \|  \|  \|  \|  \|  \|  \|  \|  \| \| +++ \|  \|  \|  \| nephritisches Syndrom \|  \| Nephritisches Syndrom [50273X\|](http://85.214.20.219/memem7/Terms_List7.cfm?search=50273X\|) \|  \|  \|  \|  \|  \|  \| \| ++ \|  \|  \|  \| nephrotisches Syndrom \|  \| Nephrotisches Syndrom [M5209\|](http://85.214.20.219/memem7/Terms_List7.cfm?search=M5209\|) \|  \|  \|  \|  \|  \|  \| \| **Physikal** \|  \|  \|  \|  \|  \|  \|  \|  \| \| **Labor** \|  \|  \|  \|  \|  \|  \|  \|  \| \| +++ \|  \|  \|  \| Autoantikörper: C3-Nephritis-Faktor \|  \| Autoantikörper [3C4\|](http://85.214.20.219/memem7/Terms_List7.cfm?search=3C4\|) C3-Nephritis-Faktor [L-1294\|](http://85.214.20.219/memem7/Terms_List7.cfm?search=L-1294\|) \|  \|  \|  \|  \|  \|  \| \| +++ \|  \|  \|  \| Komplementfaktoren: erniedrigt \|  \| Komplementfaktoren [L-2082\|](http://85.214.20.219/memem7/Terms_List7.cfm?search=L-2082\|) Erniedrigung [AD76\|](http://85.214.20.219/memem7/Terms_List7.cfm?search=AD76\|) \|  \|  \|  \|  \|  \|  \| \| +++ \|  \|  \|  \| Hämaturie \|  \| Hämaturie [M3383\|](http://85.214.20.219/memem7/Terms_List7.cfm?search=M3383\|) \|  \|  \|  \|  \|  \|  \| \| +++ \|  \|  \|  \| Proteinurie \|  \| Proteinurie [M0828\|](http://85.214.20.219/memem7/Terms_List7.cfm?search=M0828\|) \|  \|  \|  \|  \|  \|  \| \|  \|  \|  \|  \| Proteinurie \|  \| Proteinurie [M0828\|](http://85.214.20.219/memem7/Terms_List7.cfm?search=M0828\|) \|  \|  \|  \|  \|  \|  \| \|  \|  \|  \|  \| Mikrohämaturie \|  \| Mikrohämaturie [M0825\|](http://85.214.20.219/memem7/Terms_List7.cfm?search=M0825\|) \|  \|  \|  \|  \|  \|  \| \|  \|  \|  \|  \| Protein: Urin <1.5 g/Tag \|  \| Protein [L-2578\|](http://85.214.20.219/memem7/Terms_List7.cfm?search=L-2578\|) Urin [E751\|](http://85.214.20.219/memem7/Terms_List7.cfm?search=E751\|) \|  \|  \|  \|  \|  \|  \| \| +++ \| 50-100% \|  \|  \| Clinical: nephritisches Sediment: Urin \|  \| nephritisches Sediment [3442Z\|](http://85.214.20.219/memem7/Terms_List7.cfm?search=3442Z\|) Urin [E751\|](http://85.214.20.219/memem7/Terms_List7.cfm?search=E751\|) \|  \|  \|  \|  \|  \|  \| \| +++ \| 50-100% \|  \|  \| Clinical: Erniedrigung: Komplement: Serum \|  \| Erniedrigung [AD76\|](http://85.214.20.219/memem7/Terms_List7.cfm?search=AD76\|) complement [51898L\|](http://85.214.20.219/memem7/Terms_List7.cfm?search=51898L\|) Blutserum [H120\|](http://85.214.20.219/memem7/Terms_List7.cfm?search=H120\|) \|  \|  \|  \|  \|  \|  \| \| **Imaging** \|  \|  \|  \|  \|  \|  \|  \|  \| \| **Pathologie** \|  \|  \|  \|  \|  \|  \|  \|  \| \|  \|  \|  \|  \| Mikroskopie: Proliferation: Zellen: Mesangium \|  \| Zellbildung [VL\|](http://85.214.20.219/memem7/Terms_List7.cfm?search=VL\|) Körperzelle [Z0\|](http://85.214.20.219/memem7/Terms_List7.cfm?search=Z0\|) Mesangium [O4524\|](http://85.214.20.219/memem7/Terms_List7.cfm?search=O4524\|) \|  \|  \|  \|  \|  \|  \| \|  \|  \|  \|  \| Mikroskopie: Kapillaren: Mesangium: verdickt \|  \| Kapillare [T633\|](http://85.214.20.219/memem7/Terms_List7.cfm?search=T633\|) Mesangium [O4524\|](http://85.214.20.219/memem7/Terms_List7.cfm?search=O4524\|) Verbreiterung [AD73\|](http://85.214.20.219/memem7/Terms_List7.cfm?search=AD73\|) \|  \|  \|  \|  \|  \|  \| \| Lead \|  \|  \|  \| Mikroskopie: Veränderung: Basalmembran \|  \| Veränderung [AP40\|](http://85.214.20.219/memem7/Terms_List7.cfm?search=AP40\|) Basalmembran [Q9333\|](http://85.214.20.219/memem7/Terms_List7.cfm?search=Q9333\|) \|  \|  \|  \|  \|  \|  \| \| Lead \|  \|  \|  \| Mikroskopie: Proliferation: Zellen: nierenglomerulus \|  \| Zellbildung [VL\|](http://85.214.20.219/memem7/Terms_List7.cfm?search=VL\|) Körperzelle [Z0\|](http://85.214.20.219/memem7/Terms_List7.cfm?search=Z0\|) Glomerulus [O452\|](http://85.214.20.219/memem7/Terms_List7.cfm?search=O452\|) \|  \|  \|  \|  \|  \|  \| \| Lead \|  \|  \|  \| Mikroskopie: Verdickung: Basalmembran \|  \| Verbreiterung [AD73\|](http://85.214.20.219/memem7/Terms_List7.cfm?search=AD73\|) Basalmembran [Q9333\|](http://85.214.20.219/memem7/Terms_List7.cfm?search=Q9333\|) \|  \|  \|  \|  \|  \|  \| \| +++ \|  \|  \|  \| Immunhistochemie: Ablagerung: Immunglobuline: Glomerulus: subendothelial \|  \| Ablagerung [E9\|](http://85.214.20.219/memem7/Terms_List7.cfm?search=E9\|) IMMUNGLOBULINE [5046A\|](http://85.214.20.219/memem7/Terms_List7.cfm?search=5046A\|) Glomerulus [O452\|](http://85.214.20.219/memem7/Terms_List7.cfm?search=O452\|) subendothelial [3552Z\|](http://85.214.20.219/memem7/Terms_List7.cfm?search=3552Z\|) \|  \|  \|  \|  \|  \|  \| \| +++ \|  \|  \|  \| Immunhistochemie: Ablagerung: Immunglobuline: Glomerulus: Mesangium \|  \| Ablagerung [E9\|](http://85.214.20.219/memem7/Terms_List7.cfm?search=E9\|) IMMUNGLOBULINE [5046A\|](http://85.214.20.219/memem7/Terms_List7.cfm?search=5046A\|) Glomerulus [O452\|](http://85.214.20.219/memem7/Terms_List7.cfm?search=O452\|) Mesangium [O4524\|](http://85.214.20.219/memem7/Terms_List7.cfm?search=O4524\|) \|  \|  \|  \|  \|  \|  \| \| +++ \|  \|  \|  \| Immunhistochemie: Ablagerung: Immunglobuline: Glomerulus: Basalmembran \|  \| Ablagerung [E9\|](http://85.214.20.219/memem7/Terms_List7.cfm?search=E9\|) IMMUNGLOBULINE [5046A\|](http://85.214.20.219/memem7/Terms_List7.cfm?search=5046A\|) Glomerulus [O452\|](http://85.214.20.219/memem7/Terms_List7.cfm?search=O452\|) Basalmembran [Q9333\|](http://85.214.20.219/memem7/Terms_List7.cfm?search=Q9333\|) \|  \|  \|  \|  \|  \|  \| \| +++ \|  \|  \|  \| Immunhistochemie: Ablagerung: Immunglobuline: Glomerulus: subepithelial \|  \| Ablagerung [E9\|](http://85.214.20.219/memem7/Terms_List7.cfm?search=E9\|) IMMUNGLOBULINE [5046A\|](http://85.214.20.219/memem7/Terms_List7.cfm?search=5046A\|) Glomerulus [O452\|](http://85.214.20.219/memem7/Terms_List7.cfm?search=O452\|) subepithelial [3553Z\|](http://85.214.20.219/memem7/Terms_List7.cfm?search=3553Z\|) \|  \|  \|  \|  \|  \|  \| \| **Genetik** \|  \|  \|  \|  \|  \|  \|  \|  \| \| **Psychologie** \|  \|  \|  \|  \|  \|  \|  \|  \| \|  \|  \|  \|  \|  \|  \|  \|  \|  \|  \|  \|  \|  \| \| **Characteristics** \|  \| [Code](http://85.214.20.219/memem7/TermsPhemes_TransUpdate.cfm?code7=M5032\|&cl=6&concept=100C) \|  \|  \|  \|  \|  \|  \| \| **Historie** \|  \|  \| \| **Epidemologie** \|  \|  \|  \|  \|  \|  \|  \|  \| \|  \| 7-10% \|  \|  \| Prävalenz: Glomerulonephritis \|  \| Glomerulonephritis [M2059\|](http://85.214.20.219/memem7/Terms_List7.cfm?search=M2059\|) \|  \|  \|  \|  \|  \|  \| \| **Ätiologie** \|  \|  \|  \|  \|  \|  \|  \|  \| |
| --- | --- | --- | --- | --- | --- | --- | --- | --- | --- | --- | --- | --- | --- | --- | --- | --- | --- | --- | --- | --- | --- | --- | --- | --- | --- | --- | --- | --- | --- | --- | --- | --- | --- | --- | --- | --- | --- | --- | --- | --- | --- | --- | --- | --- | --- | --- | --- | --- | --- | --- | --- | --- | --- | --- | --- | --- | --- | --- | --- | --- | --- | --- | --- | --- | --- | --- | --- | --- | --- | --- | --- | --- | --- | --- | --- | --- | --- | --- | --- | --- | --- | --- | --- | --- | --- | --- | --- | --- | --- | --- | --- | --- | --- | --- | --- | --- | --- | --- | --- | --- | --- | --- | --- | --- | --- | --- | --- | --- | --- | --- | --- | --- | --- | --- | --- | --- | --- | --- | --- | --- | --- | --- | --- | --- | --- | --- | --- | --- | --- | --- | --- | --- | --- | --- | --- | --- | --- | --- | --- | --- | --- | --- | --- | --- | --- | --- | --- | --- | --- | --- | --- | --- | --- | --- | --- | --- | --- | --- | --- | --- | --- | --- | --- | --- | --- | --- | --- | --- | --- | --- | --- | --- | --- | --- | --- | --- | --- | --- | --- | --- | --- | --- | --- | --- | --- | --- | --- | --- | --- | --- | --- | --- | --- | --- | --- | --- | --- | --- | --- | --- | --- | --- | --- | --- | --- | --- | --- | --- | --- | --- | --- | --- | --- | --- | --- | --- | --- | --- | --- | --- | --- | --- | --- | --- | --- | --- | --- | --- | --- | --- | --- | --- | --- | --- | --- | --- | --- | --- | --- | --- | --- | --- | --- | --- | --- | --- | --- | --- | --- | --- | --- | --- | --- | --- | --- | --- | --- | --- | --- | --- | --- | --- | --- | --- | --- | --- | --- | --- | --- | --- | --- | --- | --- | --- | --- | --- | --- | --- | --- | --- | --- | --- | --- | --- | --- | --- | --- | --- | --- | --- | --- | --- | --- | --- | --- | --- | --- | --- | --- | --- | --- | --- | --- | --- | --- | --- | --- | --- | --- | --- | --- | --- | --- | --- | --- | --- | --- | --- | --- | --- | --- | --- | --- | --- | --- | --- | --- | --- | --- | --- | --- | --- | --- | --- | --- | --- | --- | --- | --- | --- | --- | --- | --- | --- | --- | --- | --- | --- | --- | --- | --- | --- | --- | --- | --- | --- | --- | --- | --- | --- | --- | --- | --- | --- | --- | --- | --- | --- | --- | --- | --- | --- | --- | --- | --- | --- | --- | --- | --- | --- | --- | --- |
|  |
|  |
|  |
|  |
|  |
|  |
|  |
|  |
|  |
|  |
|  |
|  |
|  |
|  |
|  |
|  |
|  |
|  |
|  |
|  |
|  |
|  |
|  |
|  |
|  |

Additional File 1: Table S8. membrano-proliferative glomerulonephritis part 2
